# Phytoestrogens and Health Effects

**DOI:** 10.3390/nu15020317

**Published:** 2023-01-09

**Authors:** Marie-Chantal Canivenc-Lavier, Catherine Bennetau-Pelissero

**Affiliations:** 1 Centre des Sciences du Goût et de l’Alimentation, CNRS, INRAE, Institut Agro, Université de Bourgogne, Franche-Comté, 21000 Dijon, France; 2 Arna U1212 Inserm, 5320 CNRS, UB Université de Bordeaux, 33000 Bordeaux, France

**Keywords:** phytoestrogens, nutrition, health, cardiovascular, metabolism, menopause, bone health, cancer, reproduction, hypothyroidism, toxicology

## Abstract

Phytoestrogens are literally estrogenic substances of plant origin. Although these substances are useful for plants in many aspects, their estrogenic properties are essentially relevant to their predators. As such, phytoestrogens can be considered to be substances potentially dedicated to plant–predator interaction. Therefore, it is not surprising to note that the word phytoestrogen comes from the early discovery of estrogenic effects in grazing animals and humans. Here, several compounds whose activities have been discovered at nutritional concentrations in animals and humans are examined. The substances analyzed belong to several chemical families, i.e., the flavanones, the coumestans, the resorcylic acid lactones, the isoflavones, and the enterolignans. Following their definition and the evocation of their role in plants, their metabolic transformations and bioavailabilities are discussed. A point is then made regarding their health effects, which can either be beneficial or adverse depending on the subject studied, the sex, the age, and the physiological status. Toxicological information is given based on official data. The effects are first presented in humans. Animal models are evoked when no data are available in humans. The effects are presented with a constant reference to doses and plausible exposure.

## 1. Introduction

Plants have been known for ages for their medicinal properties, and they are used by animals in the wild for such purposes [1,2]. Highly toxic substances are also provided by plants; curare, for instance, was used for hunting by previous Amazonian tribes [3]. Ancient pharmacopeias based on plants were developed world-wide and are still very much in use in India, Asia, South America, and in Africa. Therefore, ethnobotany is a modern science which has led to the discovery of many natural, active substances which have then been used as drugs or chemically adapted to produce new medical treatments [4]. In this context, phytoestrogens are literally plant substances with estrogenic properties that were discovered in plants in the late 19th century [5]. For a long time, they have been—and currently still are—used for their abortive actions [6,7], or for their abilities to bring relief from menopausal symptoms [8]. The main troubles associated with an estrogen deficiency at menopause are climacteric symptoms and progressive bone loss that leads to osteoporosis. The latter is a disease which brings many side consequences, including profound disabilities that can indirectly cause death. It induces large healthcare expenditures worldwide. Many studies have been performed that demonstrate the positive effects of edible phytoestrogens on climacteric symptoms, such as [9], for instance, and also on the prevention of bone loss [10]. However, recent studies have shown that phytoestrogen exposure prior to menopause can lead to adverse effects on reproductive physiology [11].

The most-studied molecules are isoflavones, since they are present in human food at active amounts. However, other compounds also produce endocrine actions. These include coumestrol, which belong to the coumestan family; 8-prenyl-naringenin, which is a prenyl-flavanone; the mycotoxins zearalenone and zearalenol, which are resorcylic acid lactones; and, finally, the enterolignans, enterodiol and enterolactone, which derive from lignans present in grains, fruits, and vegetables. Their endocrine activities rely on their affinity for estradiol receptors—both the nuclear and the membrane receptors—and also on their ability to induce estrogen-dependent gene transcription. In addition, they can also act on other cell physiological pathways that exhibit other health properties [12].

In this review, the five families of phytoestrogens are examined with respect to different health issues: these being either beneficial or adverse. Indeed, an estrogenic action can be beneficial when endogenous estrogens are missing, i.e., mainly at menopause in women. However, when estrogens are used as contraceptives, they can also be deleterious in young subjects or in those who are in a reproductive physiological status. Phytoestrogens may also be a risk when subjects face an estrogen-dependent disease. Despite numerous studies presently available in the scientific literature on phytoestrogens, controversies still remain surrounding several health endpoints. Most of them could be reasonably solved considering the concentrations and dietary dosages required. Here, therefore, the studies analyzed were essentially reporting in vivo in human beings as an attempt to determine active dosages. Phytoestrogen concentrations in food and human blood were also provided in order to check for the plausibility of dietary action and to confirm the reliability of the mechanistic data obtained in vitro.

## 2. Definition and Origin

### 2.1. Definition and Relative Potencies

**Figure 1:**
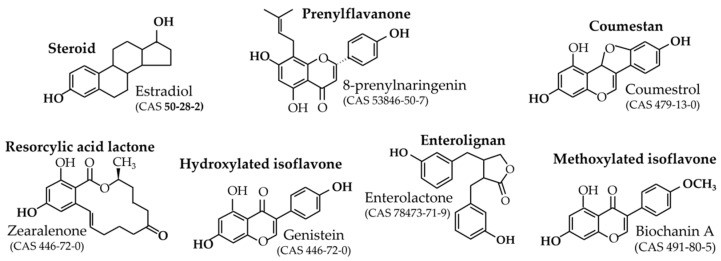
Chemical structures of the main phytoestrogens and their precursors beside the molecular structure of estradiol.

Phytoestrogens have estrogenic potencies due to their structure, which mimics that of estradiol. The common feature of all phytoestrogens, as is seen in Figure 1, is basically the presence of at least two hydroxyl functions in opposite positions on the molecule and usually at a distance of 10 angstroms, as in estradiol.

**Figure 2:**
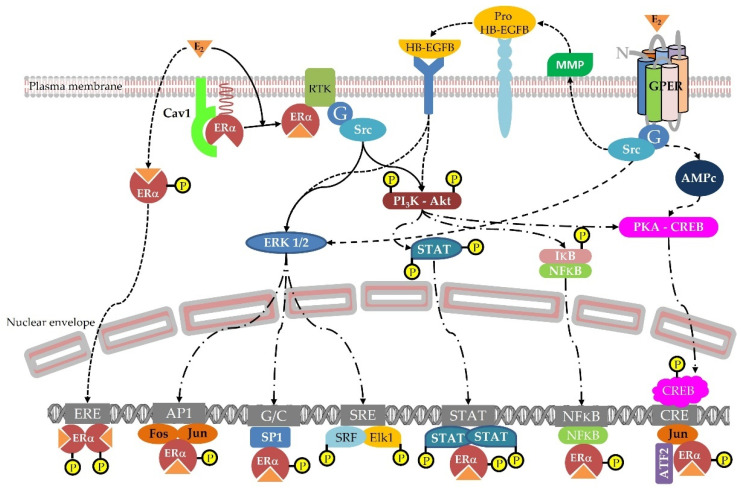
Cellular pathways triggered by estradiol via the nuclear ER, Membrane ER, and membrane GPER.

These hydroxyl groups are responsible for the interaction of phytoestrogens with the ligand binding domain of estradiol receptors [13]. Currently, three estradiol receptors are considered. The first two receptors are canonical estradiol receptors ERα and ERβ, which act mainly via a nuclear interaction with a DNA palindromic sequence called ERE [14]. However, thanks to a palmitoylation at C451A-ERα site [15], ERα can also be present just below the cell membrane, bound to caveolin, and is able to react to low concentrations of lipophilic xenoestrogens. At this location, ERα activates intracellular phosphorylation pathways that are stimulated within a few seconds, contrarily to the nuclear pathway. Several pathways have been described so far, including PI3K/Akt, Src/ERK1/2, and NFκB [16]. See Figure 2.

The third estrogen receptor is called GPER (previously GPR-30) and belongs to the rhodopsin receptor family, counting seven transmembrane domains. It is associated with G proteins bridging molecules, and it activates at least three different pathways depending on the ligand concentrations. One pathway is cAMP-dependent and reacts to 1 µM of the agonist G1, while another relies on the Src/EGFR pathways and responds to 0.01 µM of G1 [17]. Considering these different receptors, their different affinities for estrogens, their different cell locations, their different activation modes, and, finally, their different tissue distributions, it can easily be understood that the effects of estradiol—and also estrogen mimetics—can be very complex and do not follow a linear dose–response curve. Indeed, according to their specific affinities for the different receptors, some xenoestrogens exhibit bell-shaped dose–response curves, such as soy isoflavones, or U-shaped dose–response curves, such as bisphenol A. These effects can be observed at either the cell levels or the individual levels, i.e., in vivo [18]. Likewise, genistein (soy isoflavone) epigenetic effects are different according to the dose tested. In vivo, at dietary dosage in agouti mice models, genistein is a methylation activator [19], while in vitro at pharmacological doses it exerts demethylation effects [20]. In this review, effects described are preferentially reported in vivo in humans. Additionally, when animal or cell studies are described, the dosage is always mentioned and tends always to be in the physiological range.

**Figure 3:**
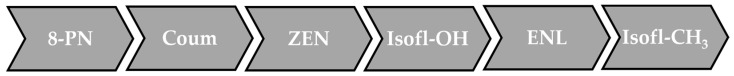
In vitro relative potencies of phytoestrogens. The scale does not take into account the metabolism and the bioavailability of the compounds. 8-PN: 8-prenylnaringenin; Coum: coumestrol; ZEN: zearalenone and zearalenol; Isofl-OH: hydroxylated isoflavones (C4′ position), i.e., genistein, daidzein, equol and glycitein; ENL: enterolignans, i.e., enterodiol and enterolactone; Isofl-CH3: methoxylated isoflavones (C4′ position), i.e., biochanin A and formononetin.

Moreover, only compounds with an estrogenic effect possibly observed in humans at plausible exposure are taken into account. This means that the present review deals with the prenyl-flavanone 8-prenylnaringenin and its precursor isoxanthohumol, coumestrol, the resorcylic acid lactones zearalenol and zearalenone, hydroxylated isoflavones genistein, daidzein, glycitein, the metabolite equol, and the enterolignans enterodiol and enterolactone. Finally, this review also concerns the methoxylated isoflavones that can be hydrolyzed into hydroxylated parent compounds. See Figure 1 for the structures of these main compounds and Figure 3 for their in vitro estrogenic potencies, sorted from the highest to the lowest.

In addition to these compounds, others have been shown to exhibit estrogenic effects in vitro or in animals when tested at pharmacological doses. These include apigenin, naringenin, quercetin, and resveratrol, etc. This review will not treat these substances.

Estradiol is essential for many different physiological functions such as reproduction, growth, food digestion and metabolism, mood expression, energy balance, core temperature management, and bone accretion, etc. Therefore, estrogens can have beneficial effects in certain physiological statuses and are used as drug-replacement therapies in several menopausal disorders. In parallel, the over-dosing of estradiol or estradiol analogues (ethynyl-estradiol, for instance) are used medically as contraceptive treatments. Therefore, the duration and the dose of estrogen exposure are crucial to consider for avoiding potential adverse effects, especially on reproduction or reproductive tissues. Finally, if Asian populations are nowadays largely exposed to some phytoestrogens, the lack of strict control populations does not allow for the deciphering of the precise consequences of such exposures.

### 2.2. Origin and Role in Plants

The different phytoestrogen compounds considered in this review can be classified into three groups:

- Mycotoxins;
- Phytoalexins;
- Non-estrogenic native compounds requiring gut-flora metabolism to become active.

This late category includes equol and enterolignans, which are estrogenic but are generated by the cooperation of human gut-bacteria clusters [21,22]. Given that not all consumers harbour the relevant bacteria species, these compounds will not be present and active in all consumers. The origin of their precursors can be named (and will be mentioned later), but ingesting these plant sources will never guarantee an exposure to estrogenic metabolites.

- Prenyl-flavanones

The prenyl-flavanones 8-prenylnaringenin, 6-prenylnaringenin, and isoxanthohumol are secreted by the lupulin glands of hops’ inflorescences. Their role in plants seems to be poorly documented, while their estrogenic activities, which are essentially provided by 8-prenylnaringenin, are often cited. However, Yan et al., [23] recently reported a significant anti-fungal activity of isoxanthohumol on *Botritis cinerea*. This means that the prenylflavanones can act as phytoalexins in hops.

- Coumestrol

Coumestrol is essentially produced in alfalfa, *Medicago sativa* (up to 36 mg/100 g Dry Mater (DM) in a Stamina 5 cultivar), and clover (14.079 mg/100 g DM), where it acts as a phytoalexin. To a lesser extent, it can also be found in mungo beans (0.932 mg/100 g DM), pinto beans (1.805 mg/100 g DM), kala chana seeds (6.130 mg/100 g DM), and in split beans (0.812 mg/100 g DM) [24]. In all cases, the pulses are considered raw, and cooking in water removes the glycosidic forms of coumestrol which are majoritarian. Such a treatment was previously used to reduce the toxic effects of alfalfa extracts on livestock reproduction [25]. In alfalfa, coumestrol is produced in response to an infestation by *Pseudopeziza medicaginis* or by *Stemphylium vesicarium* [26]. In clover, it may be present in some white clover cultivars such as Sonja, and its concentration increases when the fungus *Pythium ultimum* is inoculated [27]. It may also be present in soy; however, according to the literature, its presence is not systematic and may also be linked to a pathogenic fungus infestation. In clover and alfalfa, it is thought to play a role in the plant symbiosis with arbuscular mycorrhizae and rhizobium bacteria, which colonize plant roots to form nitrogen-fixing nodules [24,27].

- Resorcylic acid lactones

The estrogenic resorcylic acid lactones are the mycotoxins zearalenone and zearalenol types α and β. All are produced by fungi of the *Fusarium* family and develop on maturing corn, wheat, barley, rye oats, soybeans, sorghum, peanuts, and other food and feed crops, both in the field and on grains during transportation or storage. Zearalenone and zearalenol are mainly produced by *Fusarium graminearum* and *F. semitectum* [24]. Due to its structural similarity to naturally occurring estrogens, zearalenone is an estrogenic mycotoxin that induces obvious estrogenic effects in animals [28]. Zearalenone and zearalenol productions are favoured by high-humidity and low-temperature conditions. Zearalenone is stable in food under regular cooking temperature; however, it can be reduced under intense heating. In a human diet, the main sources are grain milling products, e.g., breakfast cereals, breads, and rolls. These mycotoxins are carefully managed at harvest and human exposure remains low. Efsa monitors the zearalenone and zearalenol levels in cereals and food. A tolerable daily intake (TDI) has been fixed at 0.25 µg/kg/day. This level includes zearalenone, zearalenol, and their metabolites, i.e., their glucuro- and sulfo-conjugates.

- Isoflavones

Isoflavones are present in several legumes at different concentrations. Soybeans, alfalfa, clover, and kudzu root (*Pueraria* sp.) are those containing the highest amounts of hydroxylated isoflavones or their precursors: the methoxylated isoflavones on the 4′-carbon (biochanin A and formononetin). Moreover, lentils, chickpeas, mungo beans, and broad beans contain an amount of 10 to 100 times less of isoflavones. Kudzu (*Pueraria lobata* or *P. mirifica*), a Chinese medicinal plant, also contains high amounts of genistein and daidzein in combination with puerarin. The ratio of genistein and daidzein are inverted in kudzu when compared to soy. This will be discussed later. Isoflavones also play as an attractant of arbuscular mycorrhizae and rhizobium bacteria in several pulses, including soy [24]. The contamination of a soy strain with the fungus *Diaporthe phaseolorum* f. sp. *meridionalis* induced the accumulation of isoflavones (genistein and daidzein), pterocarpans (glyceolins), and flavones (apigenin and luteolin) via the nitric oxide synthase pathway [29]. According to some authors, isoflavones also play a role as phytoalexins, preventing insect attacks in combination with UV resistance in the plant [30].

- Enterolignans

Finally, enterolactone and enterodiol are produced from seccoisolariciresinol diglucoside (SDG) by the gut flora of certain consumers [21]. Other lignans were shown to lead to estrogenic-enterolignan formation, namely, seccoisolariciresinol, lariciresinol, matairesinol, pinoresinol, syringaresinol, sesamin, sesamolin, and medioresinol [24]. Lignans are considered moieties of lignin, whose role is to provide a rigid structure to many plants, including cereals. These compounds are also present in seeds. Although lignans are known to be present in fruits and vegetables, the main source of SDG is known to be linseeds, also called flaxseeds [31].

### 2.3. Aromatic and Medicinal Plants

Certain aromatic or medicinal plants (herbal teas or essential oils) also exert endocrine effects, in particular on metabolic functions and reproductive functions. In 1975, Farnsworth reported a long list of plants that had long used in Western countries as anti-fertility agents [6,7], and 60% of them contained phytoestrogens, isoflavone-type phytoestrogens, or coumestrol. Experimental studies have identified preovulatory, pre-implantation, and post-implantation anti-fertility mechanisms induced by other plant substances affecting the hypothalamic–pituitary and female reproductive organs. Lithospermic acid, m-xylohydroquinone, coronaridine, rutin, and rottlerin also have anti-fertility properties [6,7]. However, certain volatile oils, such as quinine, castor oil, and sparteine, are considered abortifacients with toxic side effects on the foetus independent of an estrogenic mechanism [6,7,32]. Contraceptive plants contain terpenoids, alkaloids, glycosides, phenols, and other compounds which interfere with sex receptors or steroid hormones. Not all mechanisms are related to an estrogen-type mechanism; some are related to an anti-androgen-type effect. Many of them (*Polygonum hydropiper* Linn, *Citrus limonum*, *Piper nigrum* Linn, *Juniperis communis*, *Achyanthes aspera*, *Azadirachta indica*, *Tinospora cordifolia*, and *Barleria prionitis*) act by an anti-zygotic mechanism [33]. As an example, the contraceptive properties of the neem leaf oil (*Azadirachta indica*) is due to an estrogenic compound, Azadirachtin A [34], and an anti-androgenic compound that disrupts spermatogenesis in men [35].

Medicinal plants displaying estrogenic effects are used in the treatment of acute menopausal syndrome: mainly hot flushes, insomnia, vaginal atrophy, and osteoporosis, [36,37] but also mood and anxiety [38]. Estrogenic effects of aromatic or medicinal plants have often been established in clinical observations and demonstrated experimentally on the basis of plant extracts [39,40]. Therefore, further studies are required to reinforce data about their bioactive compounds beyond isoflavones, lignans, and coumestans. Fennel (*Foeniculum vulgare*), fenugreek (*Trigonella foenum-graecum*), lemon balm (*Melissa officinalis*), sage (*Salvia officinalis*), rosemary (*Rosmarinus officinalis*), and even black cohosh (*Cimifiga racemosa*) owe their estrogenic effects to polyphenols or terpenes. Thus, sage (*Saltiva officinalis*), traditionally used to suppress hot flushes and stimulate cognitive faculties in postmenopausal women, owes its effects to a glycosylated flavonoid (luteoline-7_0-glycoside) also found in other medicinal or aromatic plants (such as oregano, chasteberry *Vitex rotundifolia*, and *Vitex Agnus-castus*). The estrogenicity of terpenes and terpenoids is mediated through alpha estrogen receptors, but it is still poorly investigated [41,42]. Plants containing estrogenic terpenes and terpenoids are also used in traditional medicine in the treatment and prevention of hormonal cancers and in food supplements to correct the symptoms of menopause and/or prevent cardiovascular, immunological and/or inflammatory disorders. These molecules are particularly concentrated in the essential oils of medicinal plants including clary sage, peppermint, chamomile, and niaouli [43,44]. A recent in vitro study, having examined the estrogenic potency of several medicinal plants by using a human placenta model, points to anethole as one of the most estrogenic molecules [45]. Anethole is a phenylpropene that confers estrogenic effects to fennel and other Apiaceous plants (such as star anise and cumin) that are widely present in traditional medicine or have culinary uses in many countries as spices or seed teas; they are used for their beneficial effects on digestion, intestinal spasms, and premenstrual symptoms, or to stimulate lactation.

Fennel (*Foeniculum vulgare*) deserves special attention because of its growing consumption in different forms around the world (as a vegetable, herbal tea, or in supplements). Beneficial effects on menopausal syndrome have been established by a clinical study conducted in Iran on ninety postmenopausal women (45–60 years old): a twice-daily intake of fennel (2 × 100 mg, n = 45) for four weeks was sufficient to reduce symptoms of menopause (hot flushes, fatigue, sleep disturbances, vaginal dryness, anxiety, and irritability) vs. the placebo group (n = 45) [46]. On the other hand, a regular intake of fennel seed teas in early age in order to soothe intestinal spams occurring in babies may be a cause of premature thelarche or could advance the age of puberty in young girls [47,48,49].

Although the data relating to these plants and the molecules they contain have been expanding in recent years, they are still too incomplete or controversial to allow quantitative indications to be drawn. For this reason, this article will not focus on the phytoestrogens conveyed by medicinal plants. Nevertheless, due to their use as ingredients in nutritional supplements, the reader should be warned that the regular intake of these plants, and particularly at high dosages via dietary supplements, may lead to similar or even superior effects to those induced by the dietary intake of phytoestrogens and hence their therapeutic use [36,50,51].

## 3. Human Exposure and Bioavailability

### 3.1. Exposure According to Diet

- 8-prenylnaringenin

8-Prenylnaringenin, the most potent phytoestrogen known thus far, is exclusively present for human consumers in hoppy beers. 8-prenylnaringenin and its precursor in humans, isoxanthohumol, are also present in hops extracts used in dietary supplements for menopausal symptoms [52]. Currently, the Belgium Food Safety Agency has authorized hops extracts in food supplements only if their content of 8-prenylnaringenin and isoxanthohumol does not lead to an exposure to 8-prenylnaringenin greater than 400 µg/day for an adult [53]. According to Stevens et al. [54], 8-prenylnaringenin can be present in beer from 0.001 to 0.069 mg/L in lager and stout, respectively, Additionally, isoxanthohumol can be converted into 8-prenylnaringenin and can be present from 0.3 to 2.1 mg/L in Am. Hefeweizen and Strong ale, respectively. The bioavailability of 8-prenylnaringenin has been reported in humans in [55] and in [56]. T*max* for 8-prenylnaringenin ranges between 1 and 2 h in [55], based on an ingestion of 500 mg of 8-prenylnaringenin. Moreover, urine recovery has shown that the absorption rate ranges between 1.2 and 1.6%. In addition, in [56], where more dietary doses were used (0.25 mg 8-prenylnaringenin, 1.30 mg 6-prenylnaringenin, 0.80 mg isoxanthohumol, and 21.3 mg xanthohumol), T*max* occurred at 5.3 ± 4.5 h for 8-prenylnaringenin, and the percentage recovered in urine during the first 24 h was found to be 0.693 ± 0.597%. Therefore, although 8-prenylnaringenin is the most active phytoestrogen known so far in vitro, its bioavailability and dietary levels of exposure are low. In addition, the low T*max* prevents it from reaching a steady state level under normal conditions of beer or food-supplement consumption. However, isoxanthohumol can be converted into 8-prenyl-naringenin in people harboring the competent microflora. According to [57], the conversion rate of isoxanthohumol into 8-prenylnaringenin can vary from 0% to 46%. Currently, the proportion of 8-prenylnaringenin producers is insufficiently documented. Finally, according to [58], 8-prenylnaringenin and isoxanthohumol can act synergistically at a nanomolar range with estrogenic endocrine disruptors such as pesticides. These synergistic effects were obtained using physiologically plausible concentrations.

- Coumestrol

The coumestrol exposure in humans is hardly known. Some authors have reported that it could be present as traces in human food [24]. Coumestrol can also occur as methylated substances (4′-O-methyl and 7-O-methyl derivatives), which can be found in alfalfa [24]. When they reach the liver, the methylated forms can be demethylated into coumestrol by phase I enzymes. Coumestrol estrogenic potency is by far one of the highest in vitro, especially through ERα [59]. However, its bioavailability appears to be lower than that of isoflavones in rats [60]. As this compound is considered toxic, no data could be found on human pharmacokinetics. Therefore, bioavailability studies have not been published yet in humans.

- Resorcylic acid lactones

As has already been mentioned, zearalenol and zearalenone are mycotoxins and, as such, they are carefully monitored in human food. A recent study, led in Spain, reported the occurrence of zearalenone in food and in human urine [61]. The authors estimated the probable daily intake and compared it to the tolerable daily intake (25 µg/kg/day). They found that the estimated daily intake was higher than the tolerable daily intake, and that 10% of the urine samples analyzed had quantifiable levels of zearalenone. As zearalenone and zearalenol are toxic, their bioavailability has only been reported in rats and not in humans.

- Isoflavones

Isoflavones are currently the most present phytoestrogens in the human environment due to the adoption of vegan diets and the use of soybeans for both soy-based foodstuffs and transformed foodstuffs. While isoflavone exposure in China was recently evaluated in more than 53,000 people to range from 0.8 to 78.0 mg/day (median: 13.5 mg/day; IQR: 7.7, 21.4 mg/day) [62], the isoflavone exposure in Japan [63](estimated on more than 30,000 people) ranged from 14 to 75 mg/day. In Western countries, the isoflavone intake is currently considered to be lower: a few mg/day in the general population. However, in some subpopulations, such as vegan consumers or members of the Seventh-day Adventist Church in the U.S.A., the exposure can be higher, reaching a median of 17.9 mg/day [64]. Recently, an estimation of the French population’s exposure to soy isoflavones was published [65]. It showed that soy non-eaters consuming hidden soy through transformed foodstuffs were exposed to a mean intake of 1.9 mg/day of isoflavones, while the mean exposure among soy consumers was about 6.9 mg/day. These figures hide a great variability as, in the same recent-French-exposure estimation, soy isoflavone intake varied from 0 to 213 mg/day [65]. In [66], the authors provided an extensive set of isoflavone measurements in French foodstuffs. These data were obtained by specific ELISAs after a water extraction of the isoflavone glucosides. It can be seen that some foods can bring between 40 and 50 mg of isoflavones per usual portion. From the literature, it seems like there are no reproductive effects in adults at a dose below 20 mg/day, but an increase in hypothyroidism symptoms was noticed in women at 16 mg/day [67]. The bioavailability of soy isoflavones in men and women has been studied extensively [68], and the T*max* is known to occur usually around 8 h for the main isoflavones [69] (except equol, for which T*max* = 16 h). Such T*max* values allow isoflavones to reach a steady-state level when ingested regularly at a rhythm of twice a day [70]. In addition, according to [71], approximately 85% of the ingested isoflavones are excreted in human urine within 48 h after ingestion, showing that isoflavones are some of the most bioavailable polyphenols ever known. However, they are not available in their native form in plasma. Indeed, in soy, isoflavones are in glycosidic forms including glucoside, acetyl, and malonyl mono- or di-conjugates. On the enterocyte barrier, the lactase-phloridzine hydrolase is able to de-conjugate all polyphenols and also isoflavones. Isoflavones enter enterocytes, mainly as aglycone, where they can be re-conjugated into either glucuronide or sulfate moieties. Isoflavones, either aglucone or conjugated, are transported to the liver via the enterohepatic blood system, where metabolic transformations are further performed. Finally, only less than 5% of isoflavones are usually found as aglucone forms (active forms) in the blood stream [72] and are directly active at target cells. It should be noted that the free fraction of the native estradiol also represents a tiny proportion (<10%) of the total circulating estradiol that is usually measured. The elimination half-life of isoflavones ranges between 12 and 30 h. depending on the compound considered and recirculation phenomena between the liver—gallbladder on one hand and the duodenum on the other hand [69]. As was previously mentioned, the ingestion of clover-based dietary supplements containing formononetin and biochanin A in humans leads to an increase of daidzein and genistein in plasma. This is due to the conversion of the methoxylated isoflavones into hydroxylated parents by the CYP450 hepatic enzymes [73].

- Lignans

Finally, according to [74], the overall lignan intake in European countries was estimated to be 1.23 (0.89–1.73) mg/day. Among these substances are the enterolignan precursors. However, as was previously mentioned and is detailed below, not all consumers are able to produce high levels of the estrogenic enterolignans from their non-estrogenic parent compounds. There is a large variability in the conversion efficiency in enterolignans producers. In enterolactone producers, a great variability also exists in the conversion rate. Therefore, the bioavailability of enterolignans can only be studied in enterolignan producers. Nevertheless, even if the lignan exposure is considered to be low, in Western studies where isoflavones and lignans are measured in urine samples, enterolignans tend to be more concentrated than isoflavones [75].

### 3.2. Gut Flora Involvement

**Figure 4:**
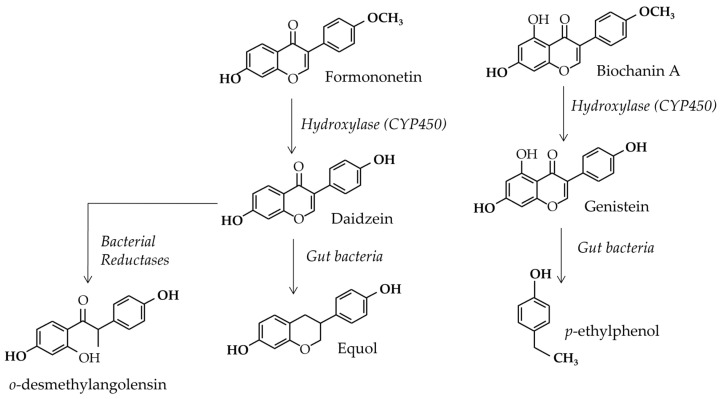
Global features of isoflavone metabolism in animals and human beings.

In humans, between 40 and 60% of people have a gut flora able to produce equol [22] from daidzein. Equol was first discovered by Marrian and Haslewood [76] in the urine of a mare. The new polyphenol was named equol for this reason and was first considered to be an inactive compound. As early as the 1960s, it was shown that gut flora was responsible for equol production [77]. Figure 4 shows the global metabolic transformations of isoflavones in animals and human beings. In humans, these conversions were recently shown to be caused by some bacterial genera [78]. Indeed, the latter were shown to be present only in equol-producing women. These are *Collinsella*, *Faecalibacterium,* and members of the *Clostridium* clusters IV and XIVa. In parallel, in [79] it was considered that the microbiological conversion of daidzein into S-equol was performed in three successive, enzymatic reactions via dihydrodaidzein and tetra-hydrodaidzein. The study reported that several equol-producing bacteria belong to diverse genera, i.e., *Eggerthella* sp. YY7918, *Lactococcus* sp. strain 20–92, *Slackia* sp. strain NATTS, and *Slackia isoflavoniconvertens*. Unlike humans, all horses and rodents (including rats and mice models), harbor an equol-producing gut flora. This means that comparing the effects of daidzein alone or in soy extracts between rodents and humans may lead to effects misinterpretation, at least for non-equol-producers. For this reason, the only isoflavone tested for toxicological effects in rats thus far is genistein. Indeed, testing daidzein in rats may lead to situations that are not easily extrapolated to humans, thus inducing potentially over-protective decisions.

**Figure 5:**
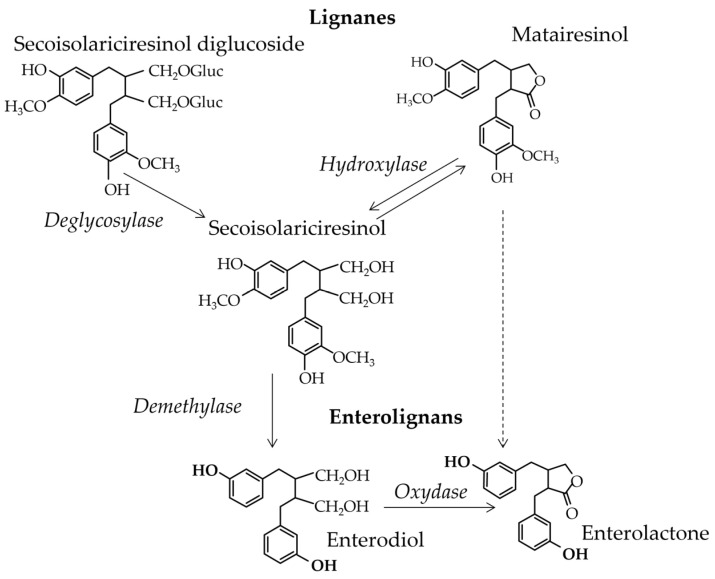
Metabolic transformation of lignans into estrogenic enterolignans by gut bacteria clusters.

Still, in humans, the enterolignans enterolactone and enterodiol are produced by specific bacteria clusters that are not present in all consumers. However, in [31], the T*max* for enterolactone was found to be observed at 24–36 h post-ingestion, while it was observed at 12 to 24 h post-ingestion for enterodiol. Such a large T*max* is explained by the gut-bacterial origin of both compounds and allows for the accumulation of both in blood, reaching a steady-state level during chronic ingestion periods. As a consequence, the level of enterolactone in human consumers’ serum and urine can vary widely, being potentially in the same range and even higher than the concentration of isoflavones, i.e., between 0.03 to 1 µM in plasma. For instance, in [80] the median serum–enterolactone concentration was 13.8 nM (range: 0–0.0956 µM) and 16.6 nM (range: 0–0.182.6 µM) in men and women, respectively. The conversion, which can be seen in Figure 5, is the metabolism of seccoisolariciresinol diglucoside into enterolactone. It is most-studied conversion thus far. This conversion requires a deglycosylation into seccoisolariciresinol and then a demethylation. The demethylation step can be undertaken by the strain *Clostridium* sp. SDGMt85-3Db, which showed the highest initial rate of deglycosylation in [81]. *Bacteroides distasonis* and *B. fragilis* were found to be predominant in enterolactone producers, even if the strain *Clostridium* sp. SDG-Mt85-3Db was also sometimes detected. Bacteria involved in the demethylation step of seccoisolariciresinol diglucoside also demethylated other lignans. In particular, *Peptostreptococcus productus* demethylated the lignans pinoresinol, lariciresinol, and matairesinol, and *Eggerthella lenta* catalysed the reduction of pinoresinol and lariciresinol into secoisolariciresinol.

Nowadays, investigations on these bacterial metabolisms are aimed at finding ways to influence the gut ecosystem for the promotion of the production of these compounds of health interest [82].

### 3.3. Blood Concentrations

As was mentioned above, this review aims to cite the plausible health effects caused by relevant doses that can lead to efficient plasma levels. This means that the blood concentrations obtained in dietary-relevant situations should be provided as a prerequisite for analyses of the effects.

- 8-prenylnaringenin

Considering 8-prenylnaringenin, serum concentrations were cited in [56] while volunteers were on a classical food-supplementation diet. The data were obtained after a single administration of 250 µg of 8-prenylnaringenin in a hops extract. Therefore, the situation represented neither chronic exposure nor a steady-state level. Nevertheless, the 8-prenylnaringenin serum concentration reached 1.4 ± 0.3 ng/mL, which represented 4.12 nM. Therefore, when in vitro effects are screened, the relevant dose should be less than 0.01 µM. This would concern circulating forms, i.e., the glucurono- or sulfo-conjugates of 8-prenylnaringenin.

- Coumestrol

Coumestrol can be found at low concentrations in the plasma of consumers. According to [83], the mean plasma level in Chinese volunteers was 2.7 ng/mL (2.2–3 ng/mL), which corresponds to 10 nM. In Mexican women [84], coumestrol–serum levels of 3 nM were described. As far as we know, there are no data on current serum measurements in European populations. An explanation for this might be that the levels are below the quantification limits of the analytical techniques. Coumestrol can be conjugated to sulfate or glucuronide residues, and the free form may even be lower than the total aglycone forms generated by the extraction procedures used in [83] and in [84].

- Resorcylic acid lactones

As was previously mentioned, zearalenone and zearalenol are mycotoxins that are carefully monitored. Therefore, the plasma levels of these mycotoxins are currently low and are below the detection limit in the majority of cases. According to [85], in which researchers analyzed several mycotoxins in the sera of 260 Chinese rural residents, “zearalanone” was detected in 1.2% of samples between 0.164 and 0.346 µg/L (0.05 and 0.1 nM) and zearalenone was detected in 6.5% of serum samples between 0.063 and 0.418 µg/L (0.02 and 0.13 nM). Although these levels were low, other mycotoxins—including aflatoxin B1, deoxynivalenol, ochratoxin A, and fumonisin—were also detected or measured, raising the question of a cocktail effect of all these substances.

- Isoflavones

Blood concentrations of isoflavones have been extensively studied. It is known that a twice-daily intake can lead to a steady-state level [70]. In this study, in which 60 menopausal women were exposed to 100 mg/day of soy isoflavones in food supplements at a rhythm of two 50 mg daily intakes (in the morning and in the evening). A steady-state concentration was obtained after five days, with a mean level of 4.56 µM eq aglycone (range: 1.61–9.96 µM). The study involved a rather high-isoflavone daily intake and could be used as an upper exposure reference. It showed that a great variability could be observed in blood concentrations, even under rigorously controlled conditions. This inter-individual variability, reaching a factor of 10, was in accordance with the toxicological safety factor that considers such a variability in the human bioavailability of xenobiotics [86]. In the study by Mathey et al. [70], the plasma–isoflavone conjugates were hydrolyzed prior to extraction and analysis. As the aglycone forms tend to represent only 5% of the total circulating compounds [72] in humans, if no conversion to aglycone forms is admitted at tissue levels, the aglycone isoflavone concentrations tested in vitro should not exceed 0.5–1 µM. If higher concentrations are tested, the effects observed can involve cell-signaling pathways that are currently not activated in vivo. Higher concentrations can only be obtained if the conversion of conjugated isoflavones into aglucone forms is demonstrated locally at tissue-level. Nevertheless, the distribution of isoflavones in tissue is generally considered to be low, confirmed by a low distribution volume. To support this property, [87] showed that, in women consuming soy milk, the genistein concentration in breast glandular tissue was three times lower than in serum (797.04 ± 237.27 pmol/mL of serum vs. 283.71 ± 35.88 pmol/g in breast glandular tissue). The study also showed that genistein in breast glandular tissue was essentially in conjugated forms: genistein-7-O-glucuronide 268 ± 179 pmol/g; genistein-4′-O-glucuronide 86 ± 46 pmol/g; and free genistein 12 ± 2 pmol/g.

- Lignans

Enterolactone levels in human blood are in the nM range. According to [84] and based on measurements performed on sixty-nine northern Mexican women, the mean enterodiol and enterolactone levels in plasma were 0.2 ± 0.6 nM and 3.8 ± 4.1 nM, respectively. Additionally, based on serum–enterolignan measurements from fifty-five Swedish menopausal women, the median plasma enterolactone level was 16.7 nM (range: 0.3–176.9 nM) [88]. Again, due to their hydroxyl groups, both enterolactone and enterodiol exist in plasma as glucuronide and sulfate conjugates [89]. Therefore, their plasma concentrations in active forms can only be 5% of their total concentrations, i.e., <10 nM. As was previously mentioned, if higher concentrations are tested in vitro, they could stimulate other cell-signaling pathways that may not be relevant in vivo.

- Methoxylated isoflavones

Finally, as has already been mentioned, the methoxylated isoflavones formononetin and biochanin A can be absorbed with some pulses, i.e., lentils, chickpeas, beans, broad beans, and mungo beans, etc. They can either be in aglucone or glucoside forms, and their glycosidic conjugates are named ononine and sissotrine, respectively. As with other glycosylated isoflavones, they can be hydrolyzed by the β-glucosidase of the enterocytes (lactase phloridzine hydrolase) and transformed into biochanin A and formononetin. Biochanin A seems to have a greater interaction with food-bowl proteins [90] and, consequently, has a lower absorption rate than its hydroxylated parent compound, genistein. Nevertheless, once the enterocyte barrier is crossed, the compounds enter the enterohepatic blood stream and are directed to the liver. There, both methoxylated compounds—biochanin A and formononetin—are metabolized by phase 1 enzymes, the so-called Cytochromes P450, which transform them into genistein and daidzein, respectively [31] (see Figure 4). Therefore, the plasma concentrations of the methoxylated isoflavones are generally below the detection limit in humans [89]. A study by Muchiri and van Breenen showed that, even with a clover formula balanced in genistein, daidzein, formononetin, and biochanin A, the resulting methoxylated isoflavone concentrations in blood were two to ten times lower than that of the hydroxylated parent compounds [91]. In the same study [91], the C*max* for formononetin in a woman ingesting 120 mg of red clover extract was 33.3 ± 7.7 ng/mL at 5 h after ingestion, while it was 8.92 ± 0.79 ng/mL under the same conditions for biochanin A. These concentrations correspond to 0.12 µM for formononetin and 0.03 µM for biochanin A. Considering the poor estrogenic potencies of the two methoxylated isoflavones, formononetin and biochanin A, the estrogenic effects of clover extract in humans should essentially rely on their hydroxylated metabolites, i.e., daidzein and genistein, respectively.

## 4. Beneficial Effects

### 4.1. Hormonal Effects

#### 4.1.1. Menopausal Symptoms

Among all the health effects of phytoestrogens that have been studied so far in humans, menopausal symptoms are the most documented. In interventional studies, no data were found on the potential effects on climacteric symptoms of menopausal and peri-menopausal women of coumestrol and resorcylic acid lactones, as they are considered to be toxic compounds. Additionally, many studies were undertaken using dietary supplements containing various classes of phytoestrogens to check for their efficiency. Currently, the market of food supplements offers preparations based on soy, clover, alfalfa, kudzu, linseed, and hops. Other plants are used for menopausal symptoms including black cohosh, chasteberry, and yam; however, their action modes are not strictly estrogenic. Although effects have been reported and confirmed by meta-analyses, the effects of phytoestrogens on menopausal symptoms are still debated. The reasons for this include a great interindividual variability, different effects according to the peri- or post-menopausal status, strong placebo effects, and studies essentially based on self-declarations.

- 8-prenylnaringenin

Hops extracts have been shown to be active on vasomotor symptoms, such as in [52]. This randomized control trial (RCT) involved 120 menopausal women and assessed the Greene score and the number of hot flushes in treated women compared to the placebo. The differences between the groups were significant after four, eight, and twelve weeks of treatment. The hops tablets used contained 500 mg of hops extract with 100 µg of the active ingredient, i.e., 8-prenylnaringenin. Although hops extracts are currently offered as food supplements for menopausal symptoms, few RCTs have been published so far.

- Isoflavones

Several meta-analyses can be cited to assess these effects. According to one meta-analysis [92], which compared several drugs and natural treatments on menopausal vasomotor symptoms, the most efficient natural treatments were those involving isoflavones and black cohosh. For isoflavones, the odd ratio and CI 95% was 0.62 (0.44–0.67) while it was 0.4 (0.16–0.90) for black cohosh.

Soy isoflavones are the most popular for menopausal-symptom relief. Another meta-analysis [93] analyzed specifically the trials involving these compounds, based on 16 articles meeting the inclusion criteria. It compared efficient values in placebo and treated groups and examined approximately 1710 subjects in total. According to this study with soy isoflavones, a 25.2% reduction in hot flushes was reported after the elimination of the placebo effect. It should be noted that this placebo effect counted for 57% of the symptom reduction by the reference treatment, i.e., estradiol. The study also showed that a time of treatment of 13.4 weeks was required for soy isoflavones to achieve half of their maximal effects. In addition, the study stated that at least 48 weeks were needed to achieve 80% of soy isoflavones’ maximum effects.

Clover treatment was not always efficient, as has been reported in the meta-analysis by Hanna and colleagues [94]. Such variable efficacy is observed at doses up to 120 mg/day.

The effect of kudzu on menopausal symptoms was studied in another meta-analysis [95]. The authors based their analysis on 8 RCTs and concluded that the efficacy of kudzu on menopausal symptoms was inconclusive. The authors pointed out methodological shortcomings, including placebo effects due to self-assessment/recall questionnaires and the lack of standardization of the kudzu extracts. They advised to improve the trials via a better knowledge of concomitant plant usages and a better assessment of the menopausal symptoms in comparison with estradiol treatments. They also recommended a pharmacovigilance assessment.

- Lignans

The effects of linseed and lignans have not been extensively studied on menopausal symptoms. In their majority, the studies are not RCTs compared to placebos and, therefore, the results are not as strong as could be expected. However, in [96], 87 women were enrolled in an RCT that compared the linseed treatment, a placebo and a soy treatment. The treatments included ground flaxseed muffins (n = 28); soy flour muffins (n = 31); and wheat flour muffins (n = 28) as the control. Hot flushes were less severe with flaxseed (*p* = 0.001) compared to the placebo, but no effect was really recorded on the other parameters measured. Similarly, a double-blind, placebo-controlled RCT [97] compared two groups of postmenopausal women: the first one consumed two slices of bread with 25 g of flaxseed (46 mg lignans) and the second group consumed wheat bran (<1 mg lignans) as a control. The treatment lasted for 12 weeks, and a similar reduction in hot-flushes frequency and the Kupperman Index were reported in both groups. Likewise, in the study by Pruthi et al. [98], an effect was recorded on peri-menopausal women showing initially at least 14 hot flushes per week and using 40 g of flaxseed flour daily for 6 weeks. A decrease was observed in hot-flush scores after flaxseed therapy (mean 57%; median 62%), from 7.3 hot flushes to 3.6 per day. However, 50% of the volunteers experienced mild or moderate abdominal distention, 29% experienced mild diarrhea, 4% experienced flatulence, and 21% withdrew because of toxicities. Indeed, 40 g of crushed linseed is quite a large dose that is able to affect gut transit. There was no placebo group in this study and therefore the effect could not be fully characterized per se. Three major reasons can explain such uncertainty in the efficacy of vasomotor-symptom treatment with lignans. (1) The enterolignan production can be highly variable from one person to another. If these enterolignans are expected to be the active compounds, a large variability in the efficacy of the treatment should be expected. (2) Lignans are commonly present in Western food and sometimes the dosages used were not high enough to induce a significant difference from the casual diet intake. (3) Menopausal symptoms are known to be sensitive to placebo effects and, according to several authors [8], a 50% reduction of vasomotor symptoms can be attributed to the placebo effect alone. Such an effect is increased since hot-flushes measurements are based on self-declarations by the volunteers.

#### 4.1.2. Bone Health

- 8-prenylnaringenin

In rats, it was shown that hops extracts standardized in 8-prenylnaringenin and administered orally could prevent bone loss without action on the rats’ uteri [99]. The efficient dose of hops extract was 60 mg/kg/bodyweight, and the serum levels of potential estrogens or precursors were: 8-prenylnaringenin—0.25 mg/kg bodyweight/day; 6-prenylnaringenin—1.31 mg/kg bodyweight/day; and isoxanthohumol—0.81 mg/kg bodyweight/day. The serum levels in treated rats were: 26.7 ± 16.9 nM, 2.9 ± 1.4 nM, and 5.8 ± 5.6 nM of 8-prenylnaringenin, 6-prenylnaringenin, and isoxanthohumol, respectively. There are no data in humans.

- Coumestrol

No data seem to exist on the effect of coumestrol on the bone health of menopausal women. Only one study tested the effect of this compound in rodents [100], showing a preventive effect against bone loss in an ovariectomized rodent model at 10 mg/kg/day. The in vivo estrogenic activity of coumestrol on bone cells has also been shown to prevent osteoclast differentiation [101] and to enhance osteoblast formation [102]. However, the doses required were from 5 to 10 µM. Such plasma concentrations can be achieved in rodents on a contaminated chow diet, but not in humans.

- Resorcylic acid lactones

Because of their toxicities, zearalenone and zearalenol were not studied for their effects on human bone health. However, some data obtained in vitro on bone cells confirmed the estrogenic effects of zearalenone and metabolites [103]. Such effects are also documented in vivo in rats or rabbits [104]. Again, the prevention of bone loss is linked to an estrogenic effect [105]. However, in some studies, chromosomal aberrations were also reported [106] and publications were included.

- Isoflavones

The main results reported on bone health concern soy isoflavones. Two meta-analyses converged in reporting that isoflavones from soy may preserve bone density in menopausal women. However, the efficiency was only observed at high doses, i.e., over 80 mg/day. The first meta-analysis [107] only considered the effect of soy-isoflavone-extract supplementation (not soy proteins) on bone mineral density (BMD) in menopausal women. It only assessed RCTs published in English, Japanese, or Chinese that reporting the effects of soy-isoflavone extracts on the lumbar spine or hip BMD in menopausal women. Eleven studies matched the fixed criteria, and the analysis included 1240 menopausal women in total. It showed that a daily intake of 82 mg of soy isoflavones (aglycone) on average, for six to twelve months, was significantly associated with a higher spine BMD. Treatment duration, geographic origin, and basal BMD were major influencing factors. There were no significant effects on either femoral neck, hip total, or trochanter BMD, and the positive effects of the soy-isoflavone-extract supplements on BMD were restricted to the lumbar spine in menopausal women. The second meta-analysis [108] included RCTs which examined the effects of soy-isoflavone supplementations in women for at least one year. The main outcomes were BMD changes from baseline at the lumbar spine, total hip, and femoral neck. A total of ten RCTs, gathering 896 women, were found to be eligible according to the retained criteria. A mean dose of 87 mg of soy isoflavones for at least one year did not significantly affect BMD. However, when doses were stratified, it was shown that only large doses—over 80 mg/day of isoflavones—tended to weakly preserve BMD at the lumbar spine and hip. Note that isoflavones have never been able to re-build an osteoporotic skeleton in humans or rodents..

- Lignans

Only few studies dealt with the association of lignans and enterolignans to bone health [109,110]. They were quite insufficient to prove any effect. The study by Dodin and colleagues [109] estimated the dietary intake of lignans and the median intake of total phytoestrogens to be 876 μg/day by using questionnaires. Such a figure is most probably underestimated, as enterolignan-precursors are significantly present in the Western diet. Nevertheless, according to the authors, the enterolignans estimated from food intake, regardless of gut-flora efficiency, were positively associated with bone density in postmenopausal women. However, this association became non-significant when dietary Ca^2+^ was added to the model. The same conclusion arose from the study by Kuhnle et al. [110]. This study estimated the phytoestrogen intake in more than 2580 women and 4930 men. It confirmed a positive association of BMD with methoxylated and hydroxylated isoflavones when Ca^2+^ was present, and also showed a positive association of enterolignan precursors with BMD. However, the latter association was no longer significant when the Ca^2+^ was taken into account. Enterolignan plasma levels should have been determined in these studies to truly help in determining their effects on the health issue examined.

#### 4.1.3. Estrogen Responsive Tissues

- 8-prenylnaringenin

There is hardly any data on hops’ phytoestrogens and breast cancer. As far as we know, the only existing data were given by Boucher and colleagues [111], who stated that the use of dietary supplements based on hops did not significantly induce breast cancer (AOR = 1.14; 95% CI = 0.36–3.56). However, the origins of these data were not clearly mentioned. In addition, although hops extracts are used in food supplements with the claim “breast enhancing,” no serious clinical study has ever been published on such an effect.

- Coumestrol

In Western developed countries, coumestrol is only anecdotally present in human food. Therefore, its effects on estrogen-dependent cancers was only scarcely examined. There are no strictly controlled RCTs, and only a few observational studies involving coumestrol are available, given that this compound is frequently under assays’ detection limits in biological samples. In [112], coumestrol, combined to precise Snp (single nucleotide polymorphism) present in the promoter region of the ERβ subtype, was shown to be strongly correlated with a decreased risk of prostate cancer. However, this correlation was weakened since the coumestrol urine levels measured in this study were close to the detection limit.

- Resorcylic acid lactones

Several data have indicated that zearalenone and its metabolites might be deleterious for breast cancers [113]. In addition, in a case-control study led in Tunisia on 110 women (69 cases and 41 control) [114], it was shown that the higher odd ratio was significantly associated with high levels of zearalenone and its metabolites in urine. Indeed, breast-cancer occurrence showed a statistically significant association with urinary concentrations of α-zearalenol (OR = 1.56; 95% CI = 1.15–2.59) in the unadjusted regression model. When the model was adjusted for age, number of children, social class, type of water consumed, and vegetable consumption, the urinary concentration of α-zearalenol remained significantly associated with breast cancer risk (OR = 1.54; 95% CI = 1.10–2.78).

- Isoflavones

As far as isoflavones are concerned, a controversy remains surrounding their beneficial and/or deleterious effects on breast cancers in women. This is due to opposite effects when cell [115] or animal [116] studies serving as toxicological references are examined, compared to epidemiological population studies [117]. If there is a clear reduction of breast cancer risk in Asian women ingesting soy from childhood [118], the preventive effect in Western women is not so obvious [119]. When metanalyses of RCTs were considered in women, the main problem was that none of them were clearly designed to see a direct effect on breast cancer. In addition, volunteers were selected based on health criteria which excluded breast cancer occurrence. Because breast cancer can occur in the general population, these studies cannot be considered to be representative of the total population. In addition, exposing women to phytoestrogens to check for an aggravation of their breast cancer is not considered ethical and is rightfully not approved by ethical committees. Because there are data arguing for both breast cancer aggravation and prevention, the best interpretation must take them all, trying to make them fit within the same mechanistical hypothesis. Through this process, it emerged that genistein may have a protective effect during the early promotion phase, i.e., when cells were healthy, while it may also act as a growth factor on estrogen-dependent tumour cells. This was in accordance with [111], which showed a reduced breast cancer risk after one year of phytoestrogen intake, suggesting that, during the first year, the tumours already present may have been boosted by phytoestrogen intakes. Once all women with undetected breast cancers had revealed their pathology, the remaining women still maintained under phytoestrogen treatments had no tumours and were protected. The protective action may be obtained via epigenetic effects, with a certain degree of transgenerational transmission. This could explain the protection observed in Asian populations exposed from childhood to modern soy foods that are rich in isoflavones [118]. Additionally, isoflavones are growth factors of breast cancer cells in vitro when they express canonical estrogen receptors or GPER [120,121]. In vitro, isoflavones doses compatible with human soy consumption exerted a growing effect. The doses of isoflavones which induced anti-proliferative effects on estrogen-dependent breast cancer cells were over 20 µM [122]. Such doses cannot be achieved physiologically. Soy isoflavones also act as growth factors in nude mice models implanted with human breast cancer cells and, in that case, the plasma levels and metabolic forms are closer to the in vivo situation in women [123]. Additionally, in the only RCT existing so far on breast cancer growth in women, plasma genistein was associated with the expression of genes controlling cell proliferation [124]. Ancient studies also showed that soy food had estrogenic and proliferative effects on healthy breast cells in premenopausal women [125]. Equally, soy isoflavones have been shown to increase the mammary density in Western, post-menopausal women [126]. To help with the interpretation of such controversial results, it should be noted that existing studies most probably underestimate soy and isoflavone intake, not considering hidden isoflavones from manufactured foodstuffs. These isoflavone levels ranged from 1.55 to 20.35 mg in Western foodstuffs containing soy [65]. Besides, in Western countries, soybean consumers tend to have healthier behaviours, which can reduce the analyses’ statistical power. Finally, when breast cancer prevalence in the West and Asia are compared, global Asian dietary habits, which include tea or fish intake besides soybean products, may also be protective [127,128].

Regarding vaginal and endometrial health, it should be reminded that both vaginal mucosa and endometria develop under estradiol stimulation and thus bear estradiol receptors: ERα, Erβ, and GPER, as well as the estradiol-related receptors ERRs [129,130,131]. The first estrogenic effect due to isoflavones ever shown was uterotrophy in New Zealand ewes grazing on clover pasture. These effects were reported either in reproductive females or in females freshly ovariectomized. In all cases, estradiol receptors were available to mediate an estrogenic effect of isoflavones. Conversely, in humans, most of the studies which were designed to show an effect of isoflavones on the genital tract were performed in menopausal women. In most studies, the time distance to menopause was largely variable and the availability of ERs in the vagina and endometrium, known to decrease after ovarian function arrest [131], was not checked. Thus, it appeared that isoflavones were globally ineffective on Western, post-menopausal women the vagina and endometrium [132]. However, in [133], a vaginal gel containing isoflavones exhibited a significant estrogenic effect on vaginal dryness, dyspareunia, and the maturing index of vaginal cells in menopausal women. In addition, in [134], which reported results obtained on Japanese students on classical Japanese diet, an isoflavone supplementation (20 or 40 mg of isoflavones daily) lengthened the menstrual cycle by two days. This lengthening of both menstruation and bleeding was dose-dependent. Considering the mean urinary levels at base line, the usual isoflavone exposure due to the Japanese diet was close to 25 mg/day. The effect appeared to vary individually and no significant modifications of either steroid or gonadotrophin hormones were noted. This suggested a direct effect of isoflavones on the endometrial mucosa. Additionally, several studies reported a potentially beneficial effect of isoflavones, particularly equol, on the prevention of premenstrual syndrome in Japanese women [135].

Considering uterine cancer; although soy isoflavones at over 20 mg/day have been shown to increase endometrial thickness [132] via endometrial cell proliferation, there are no convincing data showing a deleterious effect on endometrial cancer [136]. Indeed, it is known that hormonal replacement therapies can have opposite effects on breast and uterine cancers [137]. In the case of isoflavones, one explanation could be that the uterus, ovaries, and vagina are rich in ERβ subtypes for which isoflavones have a greater affinity than for ERα. Moreover, Erβ’s main variants are thought to be involved in cell differentiation, which counteracts the cell proliferation that is induced by Erαs [138]. To conclude, isoflavones’ effects on the vaginas and uteri of premenopausal women are not fully demonstrated and additional investigation is required. In post-menopausal women, a vaginal effect of isoflavones may be achieved, while an endometrial effect has never been clearly shown despite a large number on trials.

Concerning prostate cancer, the effects of isoflavones on the incidence of the pathology differed in Western and Asian populations [139]. The difference observed seemed to concern cancer progression, since the occurrence of cancer, as analysed by post-mortem diagnosis, showed similar frequencies between both populations [140]. Here, the type of estradiol receptors (ER) involved is crucial. Indeed, isoflavones can be protective on Erβ-bearing tumours and harmful on tumours bearing the ERβ2 variant [141,142]. They can also be anti-androgenic [143].

- Lignans

Few robust data are available so far linking the estrogenic enterolignans and estrogen-dependent diseases. This can be explained by: (1) all sources of lignans are not yet characterized in food [24]; (2) not all humans can efficiently transform lignan precursors into estrogenic enterolignans [21]; and (3) enterolignans are not usually measured in the fluids of volunteers taking part in clinical investigations.

Lignans were first correlated to a reduction of prostate cancer risk in the 1980s [144]. However, the meta-analysis by Saarinen and colleagues [145] found no correlations at the population level. A few studies involving flaxseed intake in men with a diagnosed prostate cancer did show a decrease of both serum-total PSA and the proliferation rate of benign epithelium. The authors also showed a significant decrease in total testosterone and free androgen indices [146]. According to them, enterolignans may have a protective effect on prostate cancer, but the normal dietary intake is insufficient to induce such protection. It has been more recently shown that prostate cancer pre-operating treatment with a flaxseed supplement induced the decrease of several tumour biomarkers [147]. In particular, NFκB, Ki67, and VEGF were significantly decreased, and it was concluded that flaxseed supplementation inhibits cancer cell growth and potentially reduces tumour angiogenesis in patients with prostate cancer.

Considering breast cancer, lignans and enterolignans are admitted to be protective in the global opinion [24]. Such a view is based on population studies showing that fruit and vegetable consumption globally reduces all risks of cancers, including breast cancers [148]. Although enterolignans have estrogenic properties, they can be defined as SERMs (selected estradiol receptor modulators) since they act essentially through the AF-2 transactivation domain of ERα that activates cell differentiation [149]. Conversely, and as mentioned earlier, it is the AF-1 transactivation domain which is most likely to be involved in proliferation phenomena. This mechanism was further confirmed in vivo on nude mice transplanted with MFC-7 cells [150]. In this study, dietary enterolignans were able to prevent the MCF-7 cell proliferation induced by the dietary isoflavone genistein. Regarding an interaction with GPER, no data have been found so far. In addition, studies showed that a higher intake of lignans was associated with a lower risk of developing cancer. On several occasions, the cancer’s receptor status was mentioned [151,152]. Finally, considering for isoflavones, a preventive effect in the initial phase of breast cancer progression could be retained. Indeed, in ovariectomized rats, enterolactone was shown to decrease the occurrence and size of DMBA (dimethyl benz(a)anthracen)-induced mammary tumours at a dose of 10 mg/kg/day [153]. Because DMBA was able to transform healthy mammary cells in cancer cells, the enterolactone effect could target the initial phase of cancer progression.

### 4.2. Metabolic Beneficial Effects

#### 4.2.1. Effect on Cholesterol

No data were found on the potential effect of 8-prenylnaringenin on cholesterol. In addition, there are no human data on the effects of coumestrol and zearalenone. However, an old study reported that both coumestrol and “zearanol” were able to lower cholesterol in rats [100]. In this study, the effect of genistein was much lower and the tested doses were 0.1, 1, and 10 mg/kg/day.

- Isoflavones

The effect of soybeans on cholesterol has long been studied. Soy intake was confirmed to slightly reduce both the LDL and total cholesterol (TC) in a recent meta-analysis that gathered data from 46 clinical trials [154]. The effect of soybeans in cholesterol lowering was always shown to be modest overall: LDL cholesterol was decreased by ~3.2% and TC by 2.8% when consuming ~25 g of soy protein/day. However, this lipid reduction was significant, even if heterogeneous. It is thought that the soybeans’ effect on blood lipids is due to the substitution of meat proteins containing cholesterol by pulse proteins devoid of cholesterol. To sustain this hypothesis, a similar TC-lowering effect was shown with other pulses, including peas [155]. Additionally, several studies performed on animal models [155] showed that this lipid-lowering effect could occur without phytoestrogens, as peas only contain very low levels of estrogenic isoflavones. Therefore, the role of estrogenic isoflavones in this cholesterol-lowering effect is still subjected to debate. In 1999, a RCT [156] reported that the LDL-cholesterol was isoflavone-dose-dependently reduced in subjects eating soybeans when compared to a casein control. However, the analysed subgroups counted less than 20 subjects and the overall reduction was not mentioned. Nevertheless, the highest decrease recorded in this study was 10% and was observed in the highest isoflavone-intake group (i.e., 37 mg/day). In addition, another study [157] compared the effect of soy milk proteins and water-washed soy proteins with a reduced concentration of isoflavones. No difference was observed between the groups, which counted 79 or 80 subjects exposed to the diets for three weeks. However, water washes can remove other constituents alongside isoflavones.

- Lignans

Several intervention studies gathering low numbers of subjects tended to show a lowering effect of lignan consumption on LDL or triglyceride levels. These studies monitored the enterolactone and/or enterodiol presence either in urine or in both blood and urine. Due to the low numbers of subjects and the modest effects, these studies were not always convincing [158,159]. Additionally, other population studies were performed that gave rise to contrasting results. In [160], 1492 male and female subjects of the NHANES cohort were examined for several cardiovascular risk factors and their urinary levels of enterolactone were measured. The subjects were split into three tertiles based on their urinary enterolactone levels. The study showed that enterolactone was significantly positively and negatively correlated with HDL-cholesterol and triglycerides, respectively. Additionally, LDL and TC were not significantly associated. In parallel, in a study performed by Frankenfeld [161] on 2260 subjects from the NHANES cohort, the effects of the two enterolignans were not equivalent. In this study, the population was monitored according to the fibre intake and the urinary levels of enterodiol and enterolactone. Four quartiles were defined for fibre intake as well as two levels for urinary enterolignans (high and low). The study showed that enterodiol was not associated with any of the criteria analysed. However, enterolactone urinary levels were associated with reduced obesity, reduced C-reactive protein, reduced blood pressure, reduced triglycerides, higher serum–HDL-cholesterol, and a lower occurrence of metabolic syndrome. In this study, the fibre intake was only associated with lower blood pressure and higher HDL-cholesterol. These data would sustain that enterolactone may have an effect per se. However, the differences between the two criteria considered (fibre intake and enterolactone urinary levels) may also be related to a more accurate determination of enterolignans levels than fibre intake.

#### 4.2.2. Effect on Metabolic Syndrome

- 8-prenylnaringenin

Few data exist on the regulation of glucose tolerance by prenyl-flavonoids. The only study found [162] was performed in obese mice and included xanthohumol, one precursor of 8-prenylnaringenin. This study showed that the prenylflavanones tested, which were not estrogenic, improved peripheral glucose metabolism in high-fat-diet-fed mice. However, there are no data so far on the effect of 8-prenylnaringenin itself on metabolic syndrome in either animals or humans.

- Coumestrol

Few data exist on the effect of coumestrol on metabolic syndrome and lipid-associated disorders. Data were essentially obtained on animals and express contradictory opinions. On one hand, some studies consider coumestrol as a beneficial agent for lipidic and glycemic profiles, such as [163], which examined the effect of different extracts of alfalfa sprouts in rats. In this study, 10-hydroxycoumestrol was the only polyphenol potentially involved in a beneficial effect. On the other hand, some authors considered that coumestrol, as a natural endocrine disruptor, was able to impair liver function and consequently lipid and glucose blood levels in rats [164]. Additionally, Liu et al. [83] associated plasma phytoestrogens, including coumestrol, with a lower risk of developing metabolic syndrome. However, the phytoestrogens measured in plasma could be considered biomarkers of a vegetable-based diet, which is known to reduce the risk of metabolic syndrome. Therefore, the link between coumestrol and either lipid or glucose impairments in humans is not direct and should be further demonstrated.

- Resorcylic acid lactones

There are no data exploring the effect of zearalenone on blood and lipid metabolisms in humans. However, considering the incidence of zearalenone contamination in pig farming, several studies considering the effect of zearalenone supplementation on the metabolic biomarkers in pigs were published. In [165], in which zearalenone was tested at 680 and 1620 µg/kg, the mycotoxin was found to be a naturally metabolism-disrupting chemical. It was shown to have an impact on lipid and glucose blood concentrations. The pattern was somehow complex, with a dose and time effect. Globally, lipoproteins tended to be increased by zearalenone after seven days but not after twenty-one days of treatment, and cholesterol was only increased at the lowest dose after seven days of treatment. Blood glucose was reduced at twenty-one days but increased with the low dose at seven days. The effects were only mildly significant. The authors also noted that the zearalenone altered circulating adipokine concentrations, inducing significant changes in adiponectin, resistin, and fetuin B. In [166], which tested a low exposure of zearalenone in gilt in sub-chronic exposure, i.e., 40 µg/kg/day, blood glucose was not consistently modified between the experimental and control groups over six weeks of exposure. When the experiment was designed, 40 µg/kg/day was considered to be the NOAEL, but it was reduced to 10 µg/kg/day while the experiment was ongoing.

- Isoflavones

Several reviews have addressed the link between isoflavones and metabolic syndrome. They included in vitro mechanistic demonstrations based on PPAR interactions [167] that have not always been obtained with relevant concentrations of isoflavones. Indeed, the EC_50_ of isoflavones for PPARs are usually above 20 µM, while they are below 0.1 µM for ERα and ERβ [168]. Despite this high affinity for the nuclear estradiol receptors, some authors still contest the estrogenic effects of isoflavones in vivo in humans. Therefore, considering the isoflavone–plasma levels in humans, a significant interaction of isoflavones with PPAR receptors in dietary conditions must be questioned. The following reviews can be consulted with interest [167,169,170]. However, it can be difficult to assess the specific effects of isoflavones on metabolic syndrome, as these substances are ingested at higher rate in a vegetable-based diet, which is known to reduce the risk of metabolic syndrome per se. Many studies [83,171] correlated isoflavone intake with reduced risk of metabolic symptoms. However, the effect was usually depending on sex, and such a correlation is not a definitive proof. Additionally, studies were performed based on genistein supplementation at doses of about 50 mg/day which can correspond to a dietary intake. The study [172] tested a 54 mg/day genistein supplementation on post-menopausal women with metabolic syndrome for one year. The study design was a multicentric, double-blind RCT. After one year of treatment, the treated subjects exhibited a reduced fasting glucose, fasting insulin, and HOMA-IR, while these parameters were unchanged in the placebo group. Genistein statistically increased the HDL-chol, while TC, LDL-chol, triglycerides, visfatin, and homocysteine blood levels were decreased. Systolic and diastolic blood pressures were also reduced in the treated group. Similarly, in [173], a treatment of Italian post-menopausal women with or without metabolic syndrome was performed using 54 mg genistein per day for six months. At the end of the treatment, the flow-mediated dilation (FMD) at 50 s and the peak FMD were slightly, although significantly, increased in the treated group when compared with placebo. In addition, the TC, triglycerides, homocysteine, and visfatin were significantly decreased in the genistein-treated group when compared with placebo, while blood–adiponectin levels were increased. Finally, in [174], 10 mg/day of S-Equol, a natural metabolite of daidzein, was tested on overweight and obese Japanese subjects in a double-blind, randomized, cross-over trial. There was no wash-out period, and the treatments lasted for twelve weeks. It was first shown that equol production was less frequent in the selected group (overweight and obese subjects) than in the general population, and that a supplementation with S-equol was more efficient in equol-non-producers than in equol producers. Several parameters were followed during this trial, including HbA1c, serum LDL-chol, and cardio-ankle vascular index. These parameters evolved favorably and significantly, although their modifications were tiny. The study indicated that the soy effect on metabolic syndrome may act in the sense of a reduction, and that the active compounds to look for in the diet would include genistein and also daidzein, the precursor of S-Equol.

- Lignans

Flaxseed-supplemented diets were tested several times on metabolic syndrome or some of its components, such as abdominal adiposity, waist circumference, lipid and glucose plasma levels, and insulin resistance, among others. The studies published all showed an improvement of the criteria associated with metabolic syndrome, notably in randomized clinical trials [175,176,177,178]. However, lignans are associated with fibres and fruit and vegetable intakes, which are known to reduce the risk of metabolic disorders, cardiovascular diseases, and diabetes. In addition, the ability of the gut flora to form enterolignans and especially the estrogenic enterolactone is quite variable among humans. Flaxseed contains seccoisolariciresinol di-glucoside, which can both be metabolised into enterolignans or act as such. Therefore, it is somehow uneasy to confer the effect of flaxseed to the estrogenic enterolignans themselves. The study by Frankenfeld [161], an observational study, related enterolactone–plasma levels to several criteria associated with metabolic syndrome. This study was performed on 2260 adults over 20 years old, extracted from the 2003–2010 NHANES study. The subjects were divided into two groups according to their urinary enterolactone or enterodiol levels. Only the 226 subjects with the highest and the lowest levels of enterolignans in their urine were considered for comparisons. Finally, the high urine–enterodiol concentrations were not associated with obesity or cardiometabolic risk factors. However, the high urine–enterolactone concentrations were inversely associated with obesity, abdominal obesity, high serum C-reactive protein, high serum triglycerides, low serum HDL cholesterol, and a reduced risk of metabolic syndrome. Such associations are not definitive proofs. To sustain these reservations, the randomized, clinical-controlled trial from Eriksen and colleagues [158] did not show significant effects of seccoisolariciresinol supplementation (280 mg/day) in a whole-grain rye diet when compared to a whole-grain wheat diet without supplementation. However, the monitoring of enterolactone levels in plasma and urine did show that the supplementation maintained for four weeks dramatically increased the plasma–enterolignan levels. Therefore, if an effect is to be expected, it should occur after four weeks. Regardless, these data, which were not fully conclusive, appeal to more well-designed clinical trials.

#### 4.2.3. Effects on Diabetes

- 8-Prenylnaringenin

Several data exist on the reduction of diabetic features in mice by xanthohumol and 8-prenylnaringenin [179] in previous works [180]. However, in these studies, the dosage used, i.e., 10 mg/L in the drinking water of young C57 Black-6 mice, probably exposed animals to 10 µg of 8-prenylnaringenin, which is enough to induce uterotrophy. However, none of the studies investigated this aspect, as all studies were performed on male mice. However, although the anti-diabetic effect is possible, the estrogenic effect on male reproductive features should have been investigated.

- Coumestrol

Few data can be collected when data bases are interrogated associating coumestrol and diabetes. Studies were usually performed in rodents or in vitro. In animals, they were more related to the adipocyte physiology. In vitro, the doses used in [181], between 20 and 50 µM, are far from being in line with plasma doses corresponding to human dietary exposure. Therefore, an effect of coumestrol on diabetes diseases is far from being proven.

- Resorcylic acid lactones

To our knowledge, no beneficial effects of zearalenone and zearalenol were ever reported on diabetes diseases in animals and humans.

- Isoflavones

Many studies, including epidemiological prospective cohort studies, cross-sectional studies, cases-control studies, or RCTs have been published in order to establish a definitive link between estrogenic isoflavones and Type 2 Diabetes Mellitus (T2DM). Until now, the picture has remained unclear; on one hand, data has clearly shown a beneficial effect of soy isoflavones, per se or in soy food, on the reduction of the risk of T2DM.On the other hand, more cautious studies indicate a possible link with only some isoflavones or some soy food, or in some populations, but not in others. Briefly, Tang et al. [182] analyzed 15 unique cohorts, including 565,810 individuals and 32,093 incident cases. The relative risks of developing T2DM associated with legumes or soy intake was not significant. Additionally, a risk reduction was observed with soy milk, soy proteins, tofu, and soy isoflavones. However, the authors considered that the heterogeneity between the studies was sometimes high, and that further work was needed to ascertain the risk reduction of T2DM by soy and its isoflavones. To sustain this conclusion, the study by Barańska et al. [183] showed a significant effect of soy isoflavones on lipid blood criteria, but not on blood glucose biomarkers including fasting glucose, fasting insulin, HbA1c, and HOMA-IR. This meta-analysis gathered twelve randomized controlled trials, seven parallel, randomized design trials, five case-crossover randomized design trials, and 691 subjects overall. Cautious conclusions were also drawn from the study by Glisic et al. [184]. It was performed on RCTs and nine prospective, population-based studies gathering 1687 and 212,796 subjects, respectively. The authors showed that phytoestrogen supplementation could improve fasting glucose and HOMA-IR without a significant decrease of insulin–plasma concentrations. They also showed that the results of RCTs varied with the phytoestrogen considered. Thereby, soy-derived isoflavones and genistein improved glucose homeostasis, while isoflavone mixes and daidzein had no effect or were associated with an adverse glycemic profile. The highest phytoestrogen intake was associated with a 10% risk reduction of T2DM in observational studies. The authors also mentioned that adverse glycemic profiles could be induced by soy and isoflavones in women. Conversely, in the study by Li et al. [185], which gathered eight studies, a significant inverse association was observed between soy intake and T2DM risk with a high heterogeneity. However, the relationship was obvious between the soy protein and isoflavones intake and a decreased risk of T2DM. This time, there was no heterogeneity. The protection was observed in women, in cross-sectional studies, and in Asian populations. Finally, and to highlight the complexity of the putative effects of phytoestrogens, the study by Guevara-Cruz et al. [186] showed that genistein, as a pure, supplemented compound (50 mg/day), was able to improve glucose tolerance and insulin resistance in obese subjects. In this study, all subjects exhibited strong a insulin-resistance (HOMA > 2.5) and a BMI >30. Nevertheless, after two months of genistein supplementation, the glucose tolerance was improved in the genistein-treated subjects but not in the control group. The authors showed that genistein was able to modify the gut flora, increasing, for instance, the presence of *Akkermansia muciniphila*. This bacterium has been shown to reduce obesity and insulin resistance in rodent models. The study also reported a modification of lipid metabolism that could be responsible for a change in the muscles’ lipid profile. Overall, a beneficial effect of isoflavones on T2DM seems modest and the quality of evidence remains low. The heterogeneity of the studies would indicate that isoflavones may not be the main substances responsible for the observed effects and that confounding factors still exist in these studies. All authors agreed that more high-quality evidence from prospective studies was required.

- Lignans

Several clinical studies were performed to correlate estrogenic enterolignans with health benefits. Among them was T2DM incidence. The link with enterolactone can usually be made through enterolignan measurements in plasma or urine. However, such a correlation cannot constitute a causal link, as many other substances can be found in flaxseed or other sources of lignans such as vegetables and fruits. For instance, the cross-over, randomized control trial by Pan et al. [187] was performed in 68 T2DM patients with mild hypercholesterolemia. They received a placebo or 360 mg of flaxseed lignans for twelve weeks before an eight-week wash-out and another twelve weeks of alternative treatment. The lignan supplement significantly improved HbA1c when compared to the placebo, but no significant changes were observed in fasting glucose and insulin concentrations, insulin resistance, and blood lipid profiles. Therefore, it can be said that the effect is modest. To sustain this finding, the study by Talaei et al. [188] did not show any effect of urinary phytoestrogens, isoflavones on one hand and enterolignans on the other hand, on the T2DM risk in Singaporean subjects. Briefly, the subjects were enrolled in the frame of the Singapore Chinese Health Study cohort. Initially, none of the volunteers had a T2DM profile, and they were followed for six years from 1999–2004 to 2006–2010. The subjects were sorted into four quartiles according to their initial phytoestrogen measurements in urine, and odd ratios (OR) correlating diabetes biomarkers and phytoestrogens quartiles were calculated. Whatever the phytoestrogens, i.e., genistein, daidzein, glycitein, equol, or enterodiol and enterolactone, none of the OR was significant when 95% confidence intervals were taken into account. However, lignan intake is known to be lower in Asia than in Western countries, while isoflavone intake is higher. In this context, this study shows that, in China, dietary exposure to lignans is unlikely to have an effect in consumers. Conversely, in the USA [189], where the lignan intake is higher and results in higher urine concentrations compared to the Singaporean study previously cited (2.1 nmol/mg creatinine in the urine of USA control subjects vs. 0.95 nmol/mg creatinine in the urine of Chinese control subjects), enterolactone was found to be negatively correlated with T2DM incidence. This study was a nested-case -control study based on two cohorts: the NHS and the NHSII. Enterolactone and enterodiol were measured in urine at inclusion (1995–2001) and followed until the end of 2008. The subjects were divided into four quartiles based on their enterolignan–urine levels, and the difference between the two extreme quartiles was correlated to the T2DM incidence in the two populations. Although enterolactone was significantly associated with a reduced risk of declaring T2DM, enterodiol was only marginally significantly efficient. Finally, the study by Eriksen et al. [190] was a case-control study included in a much wider cohort, i.e., the Danish Diet, Cancer, and Health cohort. This study examined the link between the enterolactone–plasma levels at pre-diagnosis and the diverse mortality causes of the subjects, including diabetes. Subjects were recruited between 1993 and 1999 and they were followed until the end of 2009. During that period, a group of 640 subjects deceased from different causes, i.e., diabetes (n = 48), cardiovascular (n = 141), cancer (n = 243), respiratory affections (n = 63), or other causes (n = 102). When plasma enterolactone was considered, four quartiles could be determined, each containing 295 or 296 people. The hazard ratios calculation, relating initial enterolactone plasma levels and death causes, showed that the risk of death was significantly decreased in the highest quartile for diabetes and cancer. This study examined for the first time the risk of death associated with enterolactone exposure. It indicated that, provided that the intake is sufficient and the follow-up duration long enough, enterolactone—not enterodiol—is likely to sign a preventive effect of diet on the incidence of T2DM. These results did not establish a definitive causal relation between enterolignans and disease occurrence.

## 5. Adverse Effects

Based on the discovery of phytoestrogens, reproductive issues will be carefully scrutinised here. However, because isoflavones are also known to interact with the thyroid function, this issue will be addressed for all phytoestrogens. In addition, in 1977, Farnsworth and colleagues reported a large list of plants that had been long used in Western countries as anti-fertility agents [6,7]. Among them, 60% contained isoflavones or coumestrol. This sustained their estrogenic effects in humans.

### 5.1. Reference Doses

When toxic effects are considered, they are usually tested in toxicological studies which are led according to validated protocols in the most appropriate model. For reproductive or cancer issues, rats or rabbits are usually used, and multigenerational exposure at low doses is usually more informative than acute exposure. Indeed, the effect of a defined compound can be different according to the period of exposure. For instance, neonatal exposure can be much more deleterious than adult exposure. However, a life-long exposure may induce physiological disorders that will be difficult to link to a define life period, especially if epigenetic effects are involved. Chronic exposure can lead to defining a lowest-observable adverse effect level (LOAEL) and a non-observable adverse effect level (NOAEL). These notions are crucial as they are used to build reference doses (RfD) that will be used as the limit of exposure for humans. Discrepancies can arise from toxicological studies, and they essentially reflect the notion of adverse effect. When the animal model used in toxicity studies shows a modification in a physiological parameter, the question is to determine if this modification is beneficial, neutral, or adverse. In the case of estrogenic endocrine disruption, the physiological criteria examined in males and/or females are usually pituitary morphology, anogenital distance, age at vaginal opening, uterotrophy, cyclicity, penis length, bulbourethral gland morphology, sperm production, fertility in first and subsequent generations, etc. In addition to these, an effect may be interpreted differently if it is observed in pups, in pregnant females, or in adults. Usually, the effects of toxic compounds are recorded and compared to each other to allow for a better transposition for human safety decision. This is the case for endocrine disruptors particularly, as their effects are usually complex and not always easy to determine. RfDs are classically derived from NOAELs, applying safety factors to transpose dose effects from rodent models to humans. The first safety factor accounts for the difference between animals and humans. It has been fixed at a value of 10 [86,191]. The second safety factor has also a value of 10 and accounts for the interindividual variation in the human species [86]. As was mentioned previously, this factor has been observed for isoflavones in human bioavailability studies [70]. Then, when a NOAEL is not available and that the only dose available is a LOAEL, a third factor is introduced as a protection for the human subjects. This factor most often equals three, but, according to some authors, it can be optimized at a lower level after appropriate statistical analysis [192]. Table 1 provides the theoretical RfD derived from the LOAEL or NOAEL available for the phytoestrogens studied here. These RfDs are compared to those of diethylstilboestrol, a synthetic estrogen considered to be endocrine disruptor, and to an estimation of the exposure evaluated in France. For lignans, the exposure is underestimated as it is only related to matairesinol intake [193]. The only compounds showing a potential intake superior to the deduced RfD in France are genistein and daidzein.

### 5.2. Hormonal Based Effects

#### 5.2.1. Pituitary Interactions

Estrogens are known to regulate pituitary reproductive hormones; namely, the follicle-stimulating hormone (FSH) and the luteinising hormone (LH). Such an effect is due to the regulation of the hypothalamic gonadotrophin-releasing hormone (GnRH) [203]. Depending on the cycle period, estradiol can either stimulate or repress pituitary hormone release. Using this property, contraceptive drugs have been developed essentially based on the synthetic ethynyl-estradiol, whose pharmacokinetic is longer than that of estradiol, conferring it a higher potency. Therefore, expecting an effect of phytoestrogens on pituitary hormone release seems sensible. If such an effect is recorded, it should induce menstrual cycle impairment and steroid synthesis modifications. In this way, phytoestrogens can act as endocrine disruptors and affect male and female fertility. As will be seen below, such effects are sometimes recorded, but other studies failed to identify any endocrine disruption. This may be due to low dosages of phytoestrogens, too short treatments, or too few tested subjects.

- 8-prenylnaringenin

As far as 8-prenylnaringenin is concerned, it is known that the estrogenic effect of hops’ inflorescences was first discovered in hops-working women who used to lick their fingers while picking up the hops buds [204]. As a consequence of this high 8-prenylnaringenin exposure, their menstrual cycles were disturbed. In rats, 8-prenylnaringenin was shown to modulate both FSH and LH release [205]. More precisely, 8-prenylnaringenin was tested at two doses (6.8 and 68.4 mg/kg bodyweight) in rats parallel to 17β-estradiol-3-benzoate (0.17 and 0.7 mg/kg bodyweight). Both doses of estradiol and the highest dose of 8-prenylnaringenin decreased serum levels of LH and FSH and increased serum prolactin levels, uterine weight, and progesterone-receptor mRNA transcripts. In the anterior pituitary, Erβ and GnRH receptor transcripts were reduced under both estradiol doses and the highest 8-prenylnaringenin dose. The mRNA concentrations of the LHα and -β subunits in the pituitary were suppressed by both estrogen treatments. These results showed that 8-prenylnaringenin had very similar, though milder, effects to estradiol on the parameters tested. However, the effective dose was high and can only correspond to the use of dietary supplements for breast enhancement. Finally, in [206] it was shown that 750 mg of 8-prenylnaringenin was able to decrease LH–serum concentration by 16.7% (95% confidence interval 0.5, 30.2) in postmenopausal women. This dose was high considering that a 50% decrease is obtained in post-menopausal women using 0.3 mg/day of ethynyl-estradiol [207]. The poor bioavailability of 8-prenylnaringenin explains this poor in vivo efficacy.

- Coumestrol

The effect of coumestrol on GnRH and LH secretion has been documented in the late 1980s [208]. In ovariectomized rats pre-treated intravenously with estradiol-17β or coumestrol, a GnRH challenge (50 ng/kg bodyweight, i.v.) was performed to check its effect on LH release. It was shown that a low-dose pre-treatment of estradiol (10 ng/kg bodyweight) significantly enhanced GnRH-induced LH release, while pre-treatment with a higher dose of estradiol (1000 ng/kg bodyweight) blocked the GnRH-induced rise. Coumestrol pre-treatment at all doses tested (10, 100, and 1000 ng/kg bodyweight) reduced without abrogating the GnRH-induced LH release. Such an experiment showed that Coumestrol in vivo can be as active as estradiol when administrated intravenously. However, in a normal situation, coumestrol should be absorbed orally and therefore its poor bioavailability would probably reduce its efficacy.

- Resorcylic acid lactones

Zearalenone and zearalenol as mycotoxins occurring in cattle diet have been shown to induce major economic losses affecting animal growth and reproduction. Therefore, they have been extensively studied in animal models, including pigs. In this species, they were shown in [209] to significantly reduce FSH synthesis and secretion. However, the resorcylic acid lactones had no effect on LH. The study also showed that both mycotoxins acted through the estrogen membrane receptor GPER. However, in the study by He et al., the doses used were huge; i.e., 7.5 mg/kg/body weight intravenously administrated. Again, a dietary exposure would most probably require higher dosages to be efficient, due to the poor bioavailability of zearalenone and zearalenol after dietary intake. In parallel, the oral administration of 10 mg/kg/body weight to rats [210] was able to increase GnRH expression in the hypothalamus and to significantly decrease the progesterone level in serum. The effects on FSH and LH were not significant.

- Isoflavones

The effects of methoxylated and hydroxylated isoflavones have been studied on animal reproduction since the discovery of the clover infertility syndrome or clover disease in the late 1940s [211]. Their effects on gonadotrophins were first reported in ewes by Findlay and colleagues [212]. Such effects were then reported in women [213] and in men [214], affecting FSH, LH and progesterone levels in pre-menopausal women [213] and affecting sperm production in men [214]. The doses required in premenopausal women were shown to be 45 mg/day in a rigorously controlled diet while, in men, the effect was reported using 120 mg/day. The disruption on menstrual cycles in women was further confirmed by other authors [134,215] and was always obtained with dosages between 40 and 50 mg/day. Such dosages can easily be achieved with the consumption of two soy-based foodstuffs per day [66]. Note that gonadotrophin hormone levels were also altered in trout fed a semi-synthetic diet enriched with 500 ppm genistein in fish farms [18]. Such a result showed that the gonadotrophin regulation by estrogen is a particularly well-conserved process among vertebrates.

- Lignans

As far as we know, there are no published data on the effect of lignans and enterolignans on human fertility, women’s menstrual cycles, and men or women’s hypothalamus and pituitary hormones.

#### 5.2.2. Estrogen Based Toxic Effects

- 8-prenylnaringenin

In humans, there are no rigorous studies showing an effect of 8-prenylnaringenin on reproduction. 8-prenylnaringenin is claimed by dietary supplement manufacturers to have breast enlargement properties. However, no scientific report exists thus far documenting such an effect in women. In addition, the lactogenic compounds in beer are most probably water-soluble β-glucans that stimulate the pituitary secretion of prolactin [216]. In [99], in which a hops extract was orally administered to rats, no effect was recorded on the uterus for a dose of 2.37 mg/kg bodyweight/day of 8-prenylnaringenin and precursors isoxanthohumol and 6-prenylnaringenin.

- Coumestrol

There is hardly any data on the effect of coumestrol on human reproduction because the common exposure is low, and when the correlation of reproductive parameters was attempted with coumestrol in biological fluids, it was unsignificant [217]. Conversely, in animals, many effects have been recorded in rats after plausible dietary administration. In [218], a review summarizing the effects of phytoestrogens neonatally administered in rodent models, it was reported that coumestrol in rats can induce early vaginal opening, increased initial uterine wet weight, and decreased adult uterine weight later. Such effects were also observed in mice neonatally treated with Diethylstilboestrol. When administered to rats during postnatal days 10–14, coumestrol reduced the number of endometrial glands observed in adults and reduced the expression of estrogen receptors. Mice treated neonatally with coumestrol also showed squamous metaplasia and an abnormal collagen deposition in the uterine wall. In adult rats, Whitten and colleagues reported various deleterious effects of coumestrol orally administered at a dose of 100 µg/g [219]. These effects were typically estrogenic and depended on the time of administration. They induced a decrease in LH production, aberrant cycles, reduced cyclicity, persistent oestrus, and uterotrophy in females. They also induced uterine cell proliferation, progestin receptor induction, estrogen receptor activation, decreased age of uterus canalization, and an early age at first oestrus. Coumestrol also affected male reproductive characters, including behaviour, by increasing the time to react to receptive females. Coumestrol also decreased testosterone serum levels and testicular size of rat exposed during adulthood, most probably via a pituitary interaction. The actual human exposure, which is generally low, would most probably avoid such endocrine disruptions.

- Resorcylic acid lactone

In a cohort of 163 girls from New Jersey, it was shown that urinary zearalenone and its metabolites were associated with slower growth and pubertal development [220]. The mycotoxins were detected in 78% of the urine samples, and the levels were at a median of 1.02 ng/mL and in a range of 0–22.3 ng/mL. Conversely, in China, Ref. [221] showed that, among many other factors including familial environment, zearalenone and its metabolites were strongly associated with idiopathic precocious puberty. This apparent discrepancy may be linked to the dose of exposure. According to a recent review [222], there are no epidemiological data on the reproductive effects of zearalenone and its metabolites. However, many data are available in rodent models, showing that at doses from 0.2 mg up to 146 mg/day the mycotoxins can alter follicular profiles in the ovaries of non-pregnant females, disrupt oestrus cycling, and increase myometrium thickness. In addition, during pregnancy, zearalenone and its metabolites are related with placental haemorrhage, stillbirth, and impaired foetal growth. In males, a recent review [223] showed that zearalenone and its metabolites increased relative epididymis weight, increased serum–estradiol levels, and decreased LH levels in prenatally exposed rats. In pubertal and adult rodents, the relative testicular weight, serum–estradiol level, Leydig cell number, and percentage of ST (+) Leydig cells decreased under zearalenone exposure. In all animals, serum testosterone levels, sperm concentration, and sperm motility rates were decreased. According to the authors, zearalenone could decrease serum testosterone levels at 50 µg/kg bodyweight/day, 1.4 mg/kg bodyweight/day, and 84 mg/kg bodyweight/day, in rodents exposed prenatally, at the onset of puberty and in adulthood, respectively. Sperm quantity and quality was impaired in rodents at puberty with 1.4 mg/kg bodyweight/day of zearalenone, while in adults the same effect was observed at the dosage of 84 mg/kg bodyweight/day. Such effects can be considered as estrogen-related toxic effects.

- Isoflavones

Many data exist on the effects of isoflavones on reproductive parameters on humans. Not all studies provided consistent or significant results, and this was essentially due to experimental bias, too short of a time of exposure, too small of a population observed, or to exposures that were below the effective dose. Indeed, unlike other endocrine disruptors that can exhibit effects at very low doses, isoflavones are substances that show a threshold effect, at least in vivo. Regarding at the literature, it can be seen that no adverse effects have ever been reported so far in humans for an exposure below 0.3 mg/kg bodyweight/day. This is 20 mg/day of aglycone hydroxylated isoflavones for an adult weighing 60 kg. Nevertheless, when compared to the phytoestrogens’ reproductive effects described for coumestrol or mycotoxins, the effects on reproduction are undoubtable based on animal-model studies. Indeed, a multigenerational reprotoxic study was published by the National Toxicology Program in the USA in 2008 [197]. It showed that females exposed to 51 mg/kg bodyweight/day of genistein gave birth to pups with: lower pre- and post-weaning weights in F0, F1, F2, F3, and F4; a reduction in anogenital distance in F1, F2, and F3; a reduction of the age at vaginal opening in F1, F2, and F3; and a cycle alteration in F1, F2, and F3. For males on 35 mg/kg bodyweight/day, the pre- and post-weaning weights were decreased in several generations. The anogenital distance was reduced in F1, and the rate of mammary gland hyperplasia was increased in F0, F1, and F2. Renal tubules showed calcifications in F1 and F2. As far as fertility was concerned, a reduction of the litter size was observed in the F2 generation. All these effects sign an estrogenic and anti-androgenic effect, as was shown for other endocrine disruptors.

In humans, breast milk from Chinese mothers was shown to contain only low levels of isoflavones compared to soy-based infant formulas. Indeed, according to Zhou et al., [224] the women’s breast milk contained average concentrations of daidzein and genistein ranging from 0.52 to 202.87 μg/kg. Additionally, according to [225], American, soy-based infant formulas contained between 32,000 and 47,000 µg/kg and infants exclusively fed these soy-based formulas were the human subjects most exposed to these substances. Thus, the neonatal exposure through soy-based infant formulas was studied in various occasions even if, in the U.S.A., safety authorities consider the adverse effects to be currently overcome by the beneficial ones [226]. From this high exposure (5 to 11 mg/kg/day according to formulas and infant age [226]), potentially leading to LH-secretion impairment, it is expected to observe a reduction of testes development at the time of exposure and a subsequent alteration of sperm production in men in adulthood. In addition, the neonatal exposure to estrogen of baby girls should result in a masculinized behaviour and an alteration of female pituitary secretions and of the genital function and physiology in adulthood. Such an impairment is suspected to lead to decreased fertility in adult women.

When differences were observed in human subjects consuming soy-based infant formula, it was generally in comparison to breastfeeding, not to cow-milk formula [227]. Likewise, in [228] it was shown that the neonatal use of soy-based infant formula reduced the testes diameter in four-month-old-babies. The exposure lasted for less than four months, and the cohort consisted only of fifteen babies in each group. However, to date, there is no investigation on the potential effect of such a neonatal exposure on sperm production and fertility in men in adulthood. Such a retrospective study would require large cohorts of volunteers, since many endocrine-disruption events could occur between early life and adulthood, potentially reducing the significance of the observations. In young girls, Adgent and colleagues showed [229] that the playing behaviour could be transiently masculinized (forty-two months old) when they had been fed soy-based infant formula during their first six months of life. In addition, several studies showed a significant increase of impaired menstrual cycles, increased menstrual bleeding and pain, and a greater incidence of uterine fibrosis in women fed soy-based infant formula during the first four to six months of their life [230,231,232,233]. Such traces of early exposure to isoflavones suggest epigenetic alterations that are now shown in girls’ vaginas [234].

Early soy exposure, i.e., in the prepubertal period, was associated by some authors with precocious puberty. In some studies, the precocious puberty incidence was increased with soy consumption in girls [235,236] and in boys [237], while in others it was decreased [238]. However, the puberty was not always assessed on the same criteria (age at menarche, breast development, facial hairs or pubarche in boys, etc.). In [239], which was performed on the Sister Study cohort, both effects were recorded. The puberty acceleration was observed in women fed infant formula between 1960–1974 and mainly in low outcome families. This suggests that the isoflavone levels that were higher in these formulas (delivering from 9 to 11 mg isoflavones/kg bodyweight/day) is important for the resulting effect. In [240], the puberty was delayed in Chinese children that were essentially breastfed during infancy but received substantial amounts of isoflavones daily during childhood. This indicates that the physiological modifications due to an exposure to significant levels of isoflavones are different according to the time of exposure (infancy or childhood).

When isoflavones are consumed by adults, high dosages can induce deleterious effects on the reproductive tract and function. Several cases of soy over-consumption were reported in men showing secondary hypogonadism, gynecomastia, and libido impairment [214,241]. In [241], the isoflavone intake was estimated at 200 mg/day (aglycone form) via 1.2 L of soy-drink/day for four years. Such a consumption led to a persistent, dramatic drop in LH and testosterone levels, explaining male behaviour impairment. Consequences of over-consumption of soy food, have also been reported in women [233,242]. In [242], the high isoflavone intake was responsible for menstrual cycle impairment under norethisterone contraception, endometrial fibrosis, uterine myomas, endometriosis features, and secondary infertility. All these pathological signs vanished when soy was stopped.

Currently, in men, five population studies linked a decrease in sperm count and quality to high isoflavones intakes (>40 mg/day) and high isoflavones levels in biological fluids [217,243,244,245,246]. Conversely, intervention studies did not show any effect, but either the time of exposure was too short [247] or the dose of exposure was below the efficient intake [248]. In American women, high isoflavone exposure (>50 mg/day) was shown to increase the occurrence of luteal-phase deficiencies that can delay conception [249] and was also shown to increase the risk of being nulliparous at the age of 26 or at menopause [64]. Such results led to a conclusion in favour of a fertility impairment in women for soy-isoflavone dosages of over 50 mg/day. In Asia, where these exposures can usually be achieved, reproductive effects of soy isoflavones are difficult to demonstrate due to the absence of control populations. Considering the epigenetic effects of isoflavones, soy arrest may not immediately lead to substantial modifications in reproductive issues when populations were exposed to high levels for two to three generations. Nevertheless, total fertility rate figures are globally lower in Asian countries than in other countries with similar contraceptive rates and a similar GDP per capita [250]. It should be noted that, at doses below 10 mg/day, isoflavones seemed to improve in vitro fertilization [251]. This sustains the threshold effect previously described.

- Lignans

Animal studies were performed to assess the reproductive toxicity of the enterolignans. In [200], the enterolignan precursor 7-hydroxymatairesinol as a potassium acetate complex was administered for days 0–21 of gestation at three different doses: 140–180, 460–740, and 1190–2930 mg/kg bodyweight/day. The highest dose was not well tolerated and induced a loss of appetite in 50% of the gestational dams. However, the study showed no effects on reproductive performances of any treatment after external, visceral, and skeletal examination of the foetuses. Based on weight alteration, the NOAEL for maternal effects was fixed at 460–740 mg/kg/bodyweight/day, whereas the NOEL for foetal development was fixed at 1190–2930 mg/kg bodyweight/day. In addition, also in rats, Colins and colleagues [252] investigated the effect of flaxseed (20 or 40%) or defatted flaxseed meal (13 or 26%) added to an AIN-93 diet on gestation only, or on gestation and maturation in a lifetime study. They showed that flaxseed did not affect foetal development but did affect indices of postnatal development, such as the oestrous cycles in females. Although several studies related urinary lignans and steroid levels in men and women, the only study which was found to deal with a reproductive-physiology effect was [253]. In this study, the authors tested a flaxseed powder including high concentrations of urinary lignans on the menstrual cycles of eighteen normally cycling women. They proceeded in a balanced, randomized, cross-over study in which each subject had their usual diet for three cycles as a control and a supplementation with flaxseed for another three cycles. The comparison was performed on the second and third cycles of each diet. Apart from anovulatory cycles, which occurred only in the control group, flax cycles were associated with a longer luteal phase (12.6 ± 0.4 vs. 11.4 ± 0.4 days; *p* = 0.002). There were no significant differences on either estradiol, estrone, DHEA-S, prolactin, or sex-hormone-binding globulin concentrations. Progesterone concentrations were not modified, but the luteal phase progesterone/estradiol ratios were higher during the flax cycles. Mid-follicular-phase testosterone concentrations were slightly higher during the flax cycles. These data were not confirmed by subsequent studies.

#### 5.2.3. Thyroid Based Toxic Effects

- 8-prenylnaringenin

No published data were retrieved on 8-prenylnaringenin and the thyroid.

- Coumestrol

Possibly due to structural similarities to thyroid hormones and to genotoxic effects, coumestrol at doses over 40µg/day were associated with higher risk of thyroid microcarcinomas in Connecticut patients taking part in a case-control study [254]. More precisely, coumestrol exposure was stratified into five levels: very low < 40 µg/day; low = 40 to 80 µg/day; medium = 80 to 130 µg/day; high = 130 to 200 µg/day and very high > 200 µg/day. The OR = 2.48, 95% confidence interval (CI) was 1.39–4.43 for low exposure; OR = 2.41, 95% CI, 1.32–4.40 for medium exposure; and OR = 2.38, 95% CI, 1.26–4.50 for very high exposure when compared to very low exposure. Interestingly the risk of thyroid cancer was not significant with exposure doses between 130 and 200 µg/day, probably due to a low number of subjects. In this study, isoflavones that were in the few-mg range were not effective or protective.

- Resorcylic acid lactone

With zearalenone and its metabolites being used in cattle as growth factors [255], there are several studies reporting thyroid dysfunction in animals under resorcylic acid lactone treatments [256]. A toxicological study was published in 1982 [257] testing 0, 0.1, 1, and 10 mg/kg bodyweight of zearalenone in Wistar rats. It showed a dose-related increase in absolute and relative thyroid gland weights in both males and females of the F0 and F1 generations. The effect was most important in F0 males. This effect on thyroid weight was probably a result of the estrogenic activity of zearalenone. However, at those dosages, no effects were observed in the ovaries, testes, uterus, seminal vesicles, and prostate gland, despite the decrease in reproductive performances.

- Isoflavones

As far as we know there are no toxicology data nor reference values for isoflavones on the thyroid function. However, interactions of soy and isoflavones on the thyroid function have been reported in hypothyroid babies since the development of soy-based infant formula in the U.S.A. in the 1960s. At that time, the condition was defined as the “soy goiter” [258], which was efficiently reduced by iodine supplementation. The article by Doerge and Sheenan [259] gives an extensive list of the existing data at the time of 2002. Other observation studies and clinical cases were reported later, such as [260,261], showing that the effect is major in hypothyroid patients and that it prevents efficient regulation by levothyroxine and can induce mental retardation in young children. The question remained for a while regarding the role of soy proteins or soy isoflavones in this adverse effect. Recently, the RCT by Sathyapalan and colleagues answered this question by testing a soy diet, bringing either 2 mg/day or 16 mg/day to lightly hypothyroid patients [67]. Although they were no statistical differences on thyroid hormone parameters between the groups exposed to the two isoflavone levels, six women evolved toward deeper hypothyroidism when fed the high-isoflavone-diet. The mechanisms by which isoflavones are involved in such a deleterious effect relies on their interactions at different steps in the thyroid endocrine system. Indeed, isoflavones decrease T3 and T4 synthesis by reducing the activity of the thyroxine peroxidase enzyme [262] and capturing iodate ions [263]. They bind to transthyretin, a thyroid-hormone blood transporter [264], and bind to the thyroid hormone receptor, being able to induce thyroid-dependent gene transcription [265] at relevant concentrations.

- Lignans

Few data are available on the influence of enterolignans on thyroid hormone levels in humans. However, a recent study [266] positively correlated higher urinary enterolactone levels with elevated TSH levels in girls who were 12–19 years of age (β = 0.196, 95% CI: 0.081, 0.311). In a group of males who were 12–19 years of age, enterodiol was significantly positively correlated with TSH and TT3 (TT3: β = 3.444, 95% CI: 0.150, 6.737; TSH: β = 0.104, 95% CI: 0.005, 0.203). Such an association would suggest an impairment of the thyroid function in humans. However, the data are too scarce to firmly conclude.

#### 5.2.4. Androgen Based Toxic Effects

As was mentioned previously, phytoestrogens are able to decrease the anogenital distance in rats. This feature is a biomarker of feminization or an anti-androgenic effect occurring in utero [267]. As far as phytoestrogens are concerned, this anti-androgenic effect most probably results from the alteration of hypothalamus and pituitary secretions of gonadotrophins during foetal development [214]. If there are many studies showing such an effect in rodent models using dietary doses of phytoestrogens such as [197,268], for instance, very little data seems to be available on the effect of phytoestrogens on the male from in utero exposure to isoflavones, although isoflavones can be identified in cord blood [269]. Rather, studies were performed looking at the effects of isoflavones on neonatal development. When such an issue was studied in humans it essentially dealt with a high neonatal exposure to soy phytoestrogens through soy-based infant formula. Finally, the results are unclear, with cases showing anti-androgenic effects [228], cases showing androgenic effects [227] and cases where no effect could be reported on androgenic biomarkers [270]. In adult men, the over-consumption of soybeans and isoflavones decreases sperm production via a dramatic drop in LH and testosterone [214]. In vitro, isoflavones only marginally bind to the androgen receptors and, in that case, do not induce gene transcription and play as anti-androgens [271]. However, it is known that aromatase, which is able to convert testosterone into estradiol as well as GPER and Erβ, for which isoflavones have a great affinity are all involved in the male-genital-tract development in humans [272]. Therefore, although the effect of isoflavones on the increased penis length in infants fed soy-based formula, as reported in [227], is not explained so far, the interaction of phytoestrogens with the androgen axis in male humans requires attention. Moreover, early heavy phytoestrogen exposure through infant formulas can definitively have an endocrine effect in male and female babies.

## 6. Taste interactions: A New Endpoints for Phytoestrogens

### 6.1. Phytostrogens and Taste Receptor

Phytoestrogens’ stereochemistry binds various cellular targets, including the G-protein-coupled taste receptors. Also called TASRs, theses taste receptors mediate sweet and umami perceptions through the TAS1R family (n = 3) and bitterness perceptions through the TAS2Rs family (n = 25). Depending on their chemical nature, polyphenols may act as activators or inhibitors of bitter taste receptors, and combinations of polyphenols (or herbal mixtures) may be used to modulate the acceptability of bitterness [273].

As a matter of fact, the polyphenolic phytoestrogens, 8-prenylnaringenin, isoflavones, equol, and coumestrol are selective agonists of the TAS2R14 and the TAS2R39 isoforms [274,275,276]. Genistein is a strong agonist of TAS2R14 and TAS2R39, with threshold values of 4 and 8 μM and EC_50_ values of 29 and 49 μM, respectively [276]. The enterolignan enterodiol is a selective antagonist of the TAS2R10 isoform. Through these means, a solution at 25 mg·L^−1^ of enterodiol (but not enterolactone) decreased by about 30% the bitterness induced by a 500 mg L^−1^ caffeine solution [277].

The TAS2R14 and TAS2R39 are also expressed in extra-oral tissues (brain, gut, airway tract, reproductive organs, etc.). The activation of TAS2R14 promotes airway-tract-cilia beat frequency and respiratory immunity and smooth muscle-cell relaxation, offering a therapeutic challenge (bronchodilatation and uterine contractions). In the intestinal tract, taste receptors mediate signalling pathways involved in anti-inflammatory processes and metabolic and dietary regulations. Both TAS2R14 and TAS2R39 are expressed in entero-endocrine cells and are involved in the regulation of entero-hormones and, consequently, in the regulation of food intake. Like other G-Protein Coupled Receptors, the activation of TAS2Rs increases GLP-1 secretion by enteroendocrine cells, and hTAS2R14 agonists, including isoflavones, induce GLP-1 secretion. Nevertheless, the hTASR39 agonists tend to increase peptide YY, but fail to reduce food intake [278]. In addition to the gastrointestinal tract, TAS2R39 also has been found in the respiratory, nervous, and reproductive systems [279]. Given that TAS2R14 is expressed in both testes and ovaries, a possible involvement in the fertility of males and females [280] has been postulated recently. Recent data regarding the beneficial/risk effects of phytoestrogens arising from an interaction with extra-oral TAS2R14 or TAS2R39 taste receptors are lacking.

### 6.2. Phytoestrogen and Taste Preference Modulation

Taste and olfactory perceptions present a sexual dimorphism which suggests the involvement of steroid hormones in their development and regulation [281]. In women, variations have been observed according to physiological status (menstrual cycle, pregnancy, menopause), with estrogens favouring the intake of salty, sweet, and fatty foods, while progesterone increases the threshold of sensitivity to bitterness, in particular during pregnancy [282]. Studies in rodents show that estrogenic and anti-androgenic compounds modulate taste preferences according to sex, age, and period of exposure [283]. Thus, several studies in rodents show that prenatal exposure to genistein at doses compatible with dietary doses (1mg/kg bodyweight/day) selectively stimulates the preference for sugar in male immature animals and not in females [284], an effect that does not persist in adulthood [285]. Additionally, in a rodent model, genistein increased salt preference in a similar manner to estradiol [286]. However, in humans, data concerning the impact of the consumption of the major phytoestrogen, i.e., isoflavones, coumestanes, and lignans, on taste perceptions are lacking. As was described above, the activation of TS2R10 by enterodiol decreases bitterness perception and, consequently, increases the tolerance to bitterness. However, with enterodiol being produced by the colon in humans, the occurrence of such an effect is rather uncertain. Knowledge about interactions of the different class of phytoestrogens must be documented. Nowadays, the bitterness of certain phytoestrogens is one of the causes of vegetable-protein aversion.

## 7. Conclusions

All phytoestrogens, as selected in this review, exhibit estrogenic activities at relevant dietary intakes. As such, they have shown beneficial effects in cases of estrogen deficiency but also potential deleterious effects when estrogens are not required. Some of the compounds described in this review are considered to be toxic compounds, such as coumestrol or zearalenol and zearalenone. 8-prenylnaringenin, the most estrogenic natural compound ever been recorded, is considered to be a pharmaceutical substance with a clear limit of use. This limit is driven by the potential aggravation of estrogen-dependent diseases. Therefore, 8-prenynaringenin can be used under medical supervision, but must be controlled in everyday life. Fortunately, except in dietary supplements, its concentration in foodstuffs (meaning beer), remains low and should not be likely to induce an estrogenic effect. The only risk might be in heavy beer drinkers, especially those who consume hoppy beers.

Isoflavones and enterolignans have a less clear status. If enterolignans cannot be found in a conventional diet and are produced by the gut flora, estrogenic isoflavones are present in modern diets, in contrast to traditional cooking and eating habits. Enterolignans, acting as SERMs, may reach active concentrations in biological fluids and may exhibit slight estrogenic effects and mainly beneficial ones. This is due to their peculiar binding to the ERα nuclear receptor and to their possible association with the prevention of T2DM and metabolic syndrome. However, isoflavones that exhibit an intermediate estrogenic activity now reach active concentrations in biological tissues and fluids. This was not the case in the past in either Western nor Asian populations. After deciphering their beneficial and adverse effects, it appears that the most plausible effects are estrogenic and anti-thyroid actions in human subjects. Beneficial effects on metabolic syndrome and diabetes are still controversial and are sometimes based on unrealistic mechanisms considering physiological blood concentrations. Beneficial effects on bone preservation and menopausal symptoms are most plausible when sufficient intake doses are involved. Meanwhile, adverse effects on reproduction, which were demonstrated on many occasions in animals, can also be observed in humans over a defined threshold.

Finally, estrogens and phytoestrogens have both beneficial and adverse effects according to the consumers’ physiological status. They should be used with discernment. Such a wise view cannot be achieved by consumers who usually do not know their exposure magnitude. Therefore, to benefit from phytoestrogens, it would be better to consider their intake via food supplements or drugs and to reduce them in conventional diets, as it was the case in earlier times.

## Figures and Tables

**Table 1:** Reference doses for the phytoestrogens studied, compared to Diethylstilbestrol as a reference and to the potential intake as recorded in France.

Compounds	Chemical Family	NOAEL or LOAEL * in Animal	Model Species	Theoretical Reference Dose for Human ** (RfD)	Potential Intake in France	References
Diethylstilbestrol	E2 analogue	NOAEL 5 mg/kg/d	Rat	0.05 mg/kg bw/d	Drug forbidden	[194]
8-prenylnaringenin	Phytoestrogen	-	Human	400 µg/d *	No data available	[53]
Coumestrol	Phytoestrogen	LOAEL 100 mg/kg/d	Mouse	0.33 mg/kg bw/d	0.016 µg/kg bw/d	[193,195]
Zearalenone	Mycotoxin	NOAEL 40 µg/kg/d	Pig	0.4 µg/kg bw/d	0.042 µg/kg bw/d	[193,196]
Genistein	Phytoestrogen	LOAEL 35 mg/kg/d	Rat	0.12 mg/kg bw/d	0–1.5 mg/kg bw/d	[197,198]
Daidzein	Phytoestrogen	NOAEL 50 mg/kg/d	Hen	0.5 mg/kg bw/d	0–0.8 mg/kg bw/d	[198,199]
Enterolactone	Phytoestrogen	NOAEL 7-hydroxymatairesinol 460–740 mg/kg/d	Rat	4.6–7.4 mg/kg bw/d	1.64–18.2 µg/kg bw/d matairesinol	[193,200]
Biochanin A	Phytoestrogen	LOAEL 25 mg/kg/d	Rat	0.083 mg/kg bw/d	0.0003 mg/kg bw/d	[193,201]
Formononetin	Phytoestrogen	NOAEL 5 mg/kg/d	Mouse	0.05 mg/kg bw/d	0.0013 mg/kg bw/d	[193,202]

* the value is the limit fixed for human adults by the Belgian health authorities. ** Safety factor from LOAEL to NOAEL = 3 in this table.

## Data Availability

This review gather references to previous articles. When available doi identifiers were provided allowing the reader to consult the initial data.
